# Determining the Effect of Natural Selection on Linked Neutral Divergence across Species

**DOI:** 10.1371/journal.pgen.1006199

**Published:** 2016-08-10

**Authors:** Tanya N. Phung, Christian D. Huber, Kirk E. Lohmueller

**Affiliations:** 1 Interdepartmental Program in Bioinformatics, University of California, Los Angeles, Los Angeles, California, United States of America; 2 Department of Ecology and Evolutionary Biology, University of California, Los Angeles, Los Angeles, California, United States of America; 3 Department of Human Genetics, David Geffen School of Medicine, University of California, Los Angeles, Los Angeles, California, United States of America; University of Washington, UNITED STATES

## Abstract

A major goal in evolutionary biology is to understand how natural selection has shaped patterns of genetic variation across genomes. Studies in a variety of species have shown that neutral genetic diversity (intra-species differences) has been reduced at sites linked to those under direct selection. However, the effect of linked selection on neutral sequence divergence (inter-species differences) remains ambiguous. While empirical studies have reported correlations between divergence and recombination, which is interpreted as evidence for natural selection reducing linked neutral divergence, theory argues otherwise, especially for species that have diverged long ago. Here we address these outstanding issues by examining whether natural selection can affect divergence between both closely and distantly related species. We show that neutral divergence between closely related species (e.g. human-primate) is negatively correlated with functional content and positively correlated with human recombination rate. We also find that neutral divergence between distantly related species (e.g. human-rodent) is negatively correlated with functional content and positively correlated with estimates of background selection from primates. These patterns persist after accounting for the confounding factors of hypermutable CpG sites, GC content, and biased gene conversion. Coalescent models indicate that even when the contribution of ancestral polymorphism to divergence is small, background selection in the ancestral population can still explain a large proportion of the variance in divergence across the genome, generating the observed correlations. Our findings reveal that, contrary to previous intuition, natural selection can indirectly affect linked neutral divergence between both closely and distantly related species. Though we cannot formally exclude the possibility that the direct effects of purifying selection drive some of these patterns, such a scenario would be possible only if more of the genome is under purifying selection than currently believed. Our work has implications for understanding the evolution of genomes and interpreting patterns of genetic variation.

## Author Summary

Genetic variation at neutral sites can be reduced through linkage to nearby selected sites. This pattern has been used to show the widespread effects of natural selection at shaping patterns of genetic diversity across genomes from a variety of species. However, it is not entirely clear whether natural selection has an effect on neutral divergence between species. Here we show that putatively neutral divergence between closely related species (human and chimp) and between distantly related pairs of species (humans and mice) show signatures consistent with having been affected by linkage to selected sites. Further, our theoretical models and simulations show that natural selection indirectly affecting linked neutral sites can generate these patterns. Unless substantially more of the genome is under the direct effects of purifying selection than currently believed, our results argue that natural selection has played an important role in shaping variation in levels of putatively neutral sequence divergence across the genome. Our findings further suggest that divergence-based estimates of neutral mutation rate variation across the genome as well as certain estimators of population history may be confounded by linkage to selected sites.

## Introduction

Determining the evolutionary forces affecting genetic variation has been a central goal in population genetics over the past several decades. A large body of empirical and theoretical work has suggested that neutral genetic variation within a species (diversity) can be influenced by nearby genetic variants that are affected by natural selection (reviewed in [1]). This can occur via two mechanisms. In a selective sweep, a neutral allele linked to a beneficial mutation will reach high frequency [2,3]. Selective sweeps reduce neutral genetic variation near regions of the genome that are directly affected by natural selection. The second process, background selection, also reduces neutral genetic variation [4–7]. Here, purifying selection that eliminates deleterious mutations also removes nearby neutral genetic variation. Many empirical studies have found strong evidence for the effects of background selection and selective sweeps affecting patterns of neutral genetic diversity (intra-species DNA differences) across the human genome. For example, several studies have reported a correlation between genetic diversity and recombination rate [8–13]. This correlation can be driven by selective sweeps and background selection because these processes affect a larger number of base pairs in areas of the genome with a low recombination rate than with a high recombination rate. Additionally, other studies found reduced neutral genetic diversity surrounding genes [12–17], which is consistent with the idea that there is more selection occurring near functional elements of the genome.

While the evidence for natural selection reducing genetic diversity at linked neutral sites is unequivocal, the effect of natural selection on linked neutral divergence between species (inter-species DNA differences) is less clear. Elegant theoretical arguments have suggested selection does not affect the substitution rate at linked neutral sites [18,19]. However, these theoretical arguments do not include mutations that arose in the common ancestral population, the population that existed prior to the split and formation of two descendant lineages. Such ancestral polymorphism has been shown to be a significant confounder in estimating population divergence times [20]. When also including ancestral polymorphism, it becomes less clear whether selection affects divergence at linked neutral sites.

**Fig 1:**
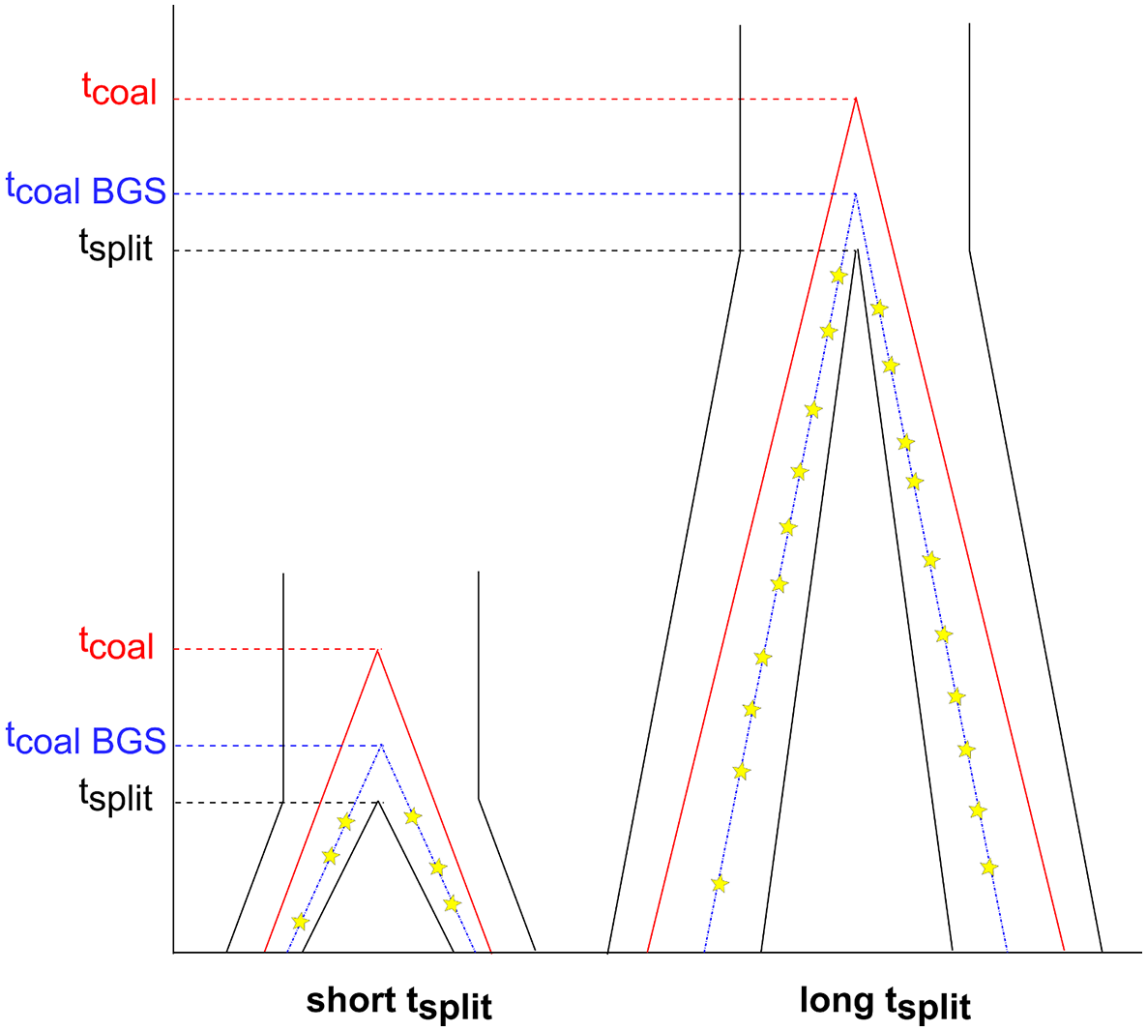
Models of how genealogies are affected by selection at linked neutral sites. The genealogies on the left represent species with a short split time such as human and chimpanzee. The genealogies on the right represent species with a long split time such as human and mouse. Red lines represent two lineages and their coalescent time. Blue lines represent two lineages and their coalescent time when there is selection at linked neutral sites in the ancestral population (abbreviated BGS). Yellow stars denote mutations accumulating on each of the two lineages after they split. Note that with the longer split time, the proportion of the genealogy attributed to the ancestral population decreases.

Based on coalescent arguments, neutral polymorphism in the ancestral population will be affected by linkage to selected sites the same way as genetic diversity within a population (Fig 1). Presumably, neutral divergence between closely related species, with lots of ancestral polymorphism, could be affected by selection. Indeed, McVicker et al. [15] demonstrated that background selection could explain the variation in human-chimp neutral divergence across the genome. Additionally, Cruickshank and Hahn [21] found that divergence between recently separated species pairs was reduced in regions of low recombination and in “islands of speciation”. They attributed at least some of these patterns to selection affecting linked neutral sites.

However, the reduction in neutral diversity in the ancestral population is thought to have a negligible effect and/or be undetectable when considering neutral divergence from species with a very long divergence time [9,18] because there would be many opportunities for mutations to occur after the two lineages split (Fig 1). These neutral mutations that occur after the split would not be influenced by selection at linked neutral sites [18] and would dilute the signal from the ancestral polymorphism. Thus, it is generally believed that selection at linked neutral sites should not affect divergence between distantly related species. An example of this argument was presented by Hellmann et al. [9]. They argued that the positive correlation between human-baboon divergence and human recombination was due to mutagenic recombination, rather than selection affecting linked neutral sites, because of the long split time between humans and baboons (>20 million years). Reed et al. [22] suggested that though it is unlikely background selection by itself could explain the entire correlation observed by Hellmann et al., background selection may still contribute to divergence. However, there has been little quantitative investigation of the effect that selection has on divergence at linked neutral sites among distantly divergent species when including ancestral polymorphism.

In addition to conflicting conceptual predictions about the expected effect of selection on divergence at linked neutral sites, empirical studies also have been ambiguous. While some studies found no evidence for a correlation between divergence and recombination such as in *Drosophila* [23,24] or in yeast [25], other studies have reported correlations between divergence and recombination in *Drosophila* [26,27]. Further, positive correlations between human-chimpanzee divergence and human recombination rate [10,12,13], human-macaque divergence and human female recombination rate [28], or human-baboon divergence and human recombination rate [9] have been reported. Finally, even though there was evidence for a strong reduction in human-chimpanzee divergence and human-macaque divergence surrounding genes [15,28], McVicker et al. [15] attributed the reductions seen for human-dog divergence to variation in mutation rates. Thus, the degree to which divergence is affected by selection across species with different split times remains elusive.

Determining whether and how selection affects linked neutral divergence is critical to understanding the evolutionary forces influencing genetic variation and mutational processes. If selection in the ancestral population only has a limited effect on divergence, it would suggest correlations between recombination and divergence to be evidence of mutagenic recombination. This may further suggest the need to consider recombination rates when modeling variation in mutation rates across the genome [9,29–32]. Because mutations rates have been difficult to estimate reliably in humans [33,34], understanding the biological factors influencing them will be of paramount importance for obtaining improved estimates. If, on the other hand, selection can affect linked neutral divergence, reductions of linked neutral divergence surrounding genes would suggest an abundance of selection affecting linked neutral sites [35]. Selection affecting linked neutral diversity and divergence is at odds with the neutral and nearly neutral theories [36–38], which have been the prevailing views in molecular population genetics for the last several decades. It would also suggest the need to consider the effects of selection when estimating mutation rates from neutral divergence.

Here we aim to examine the effects of selection on linked neutral divergence for pairs of species with a range of split times. We first present evidence that neutral divergence is reduced at putatively neutral sites close to selected sites across a wide range of taxa, including those with split times as long as 75 million years ago. Factors such as hypermutable CpG sites, GC content, or biased gene conversion by themselves cannot explain these results. We then use coalescent simulations to explore whether models incorporating background selection in the ancestral population could generate the empirical patterns. We also present a theoretical argument as to how background selection can affect variation in neutral divergence across the genome, even for species with a long split time such as human and mouse. Finally, we show that purifying selection directly reducing divergence at putatively neutral sites cannot explain these findings unless a large fraction of the genome is directly under selection, or there is a substantial number of sites under selection in the human or mouse lineage that are not conserved across species. Even though we cannot formally reject the direct effects of purifying selection from driving some of these correlations, our empirical and simulation-based findings indicate that natural selection can indirectly affect neutral genetic divergence. In sum, the view that selection does not affect divergence at linked neutral sites between distantly diverged species should be re-considered.

## Results

### Obtaining putatively neutral divergence

We wished to test whether the genetic divergence at a linked neutral site is influenced by the indirect effects of natural selection. As such, we set out to obtain putatively neutral sites by removing sites that were potentially functional and under the direct effects of purifying selection. In particular, a site was considered putatively neutral if it was (1) located at least 5kb from the starting or ending position of an exon, (2) not located within a phastCons element that was calculated over different phylogenic scopes, (3) not alignable between human and zebrafish, and (4) not found within the top 10% of most conserved Genomic Evolutionary Rate Profiling (GERP) scores [39]. Criteria 2 and 3 remove sites that are likely to be conserved across species and therefore not neutral. We chose these filtering criteria following previous studies [12,13,40]. Additionally, we chose to remove the top 10% of sites having the most extreme GERP scores because previous work suggests <10% of the genome was under the direct effect of selection [39,41–49]. The putatively neutral sites close to genes show comparable levels of divergence to four-fold degenerate sites (S1 Fig, S1 Table). As four-fold degenerate sites are often used as a neutral standard in molecular evolution, the fact that they show similar levels of divergence as our putatively neutral noncoding sites argues that our putatively neutral sites are unlikely to be under additional direct effects of selection.

### Effects on putatively neutral divergence between humans and primates

**Fig 2:**
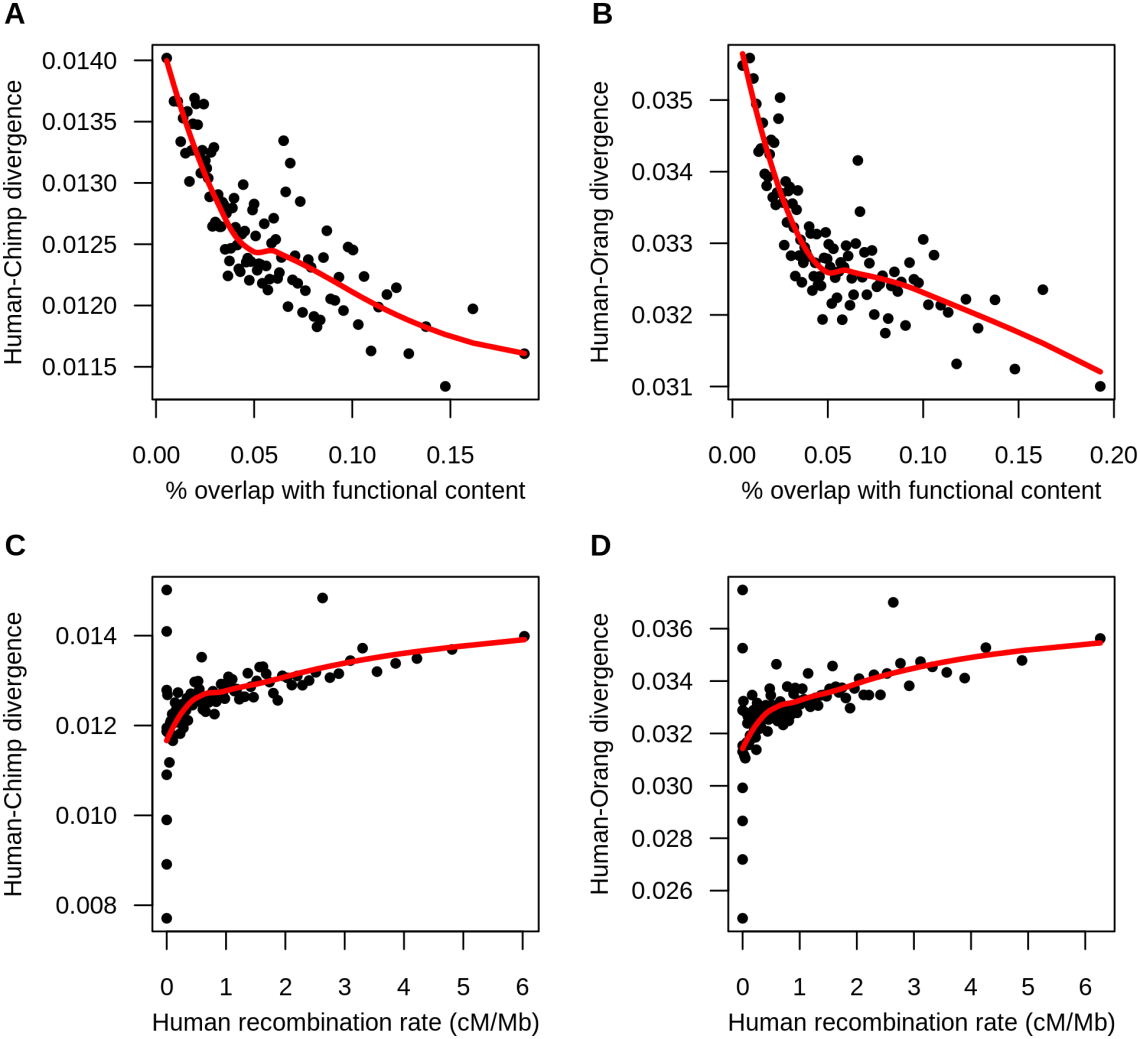
Human-primate divergence is reduced at putatively neutral sites near selected sites. (A) Neutral human-chimp divergence is negatively correlated with functional content. (B) Neutral human-orang divergence is negatively correlated with functional content. (C) Neutral human-chimp divergence is positively correlated with human recombination rate. (D) Neutral human-orang divergence is positively correlated with human recombination rate. Each point represents the mean divergence and functional content (A and B) or recombination rate (C and D) in 1% of the 100kb windows binned by functional content or recombination rate. Red lines indicate the loess curves fit to divergence and functional content (A and B) and divergence and recombination rate (C and D). The high variance of divergence at regions of low recombination rate is expected since the variance of divergence is inversely proportional to the recombination rate. Note that the last bin containing less than 1% of the windows was omitted from the plot. While the graph presents binned data, the correlations reported in the text are from the unbinned data.

To understand the evolutionary factors affecting linked neutral divergence between closely related species, we examined human-primate divergence, particularly human-chimp divergence and human-orangutan divergence. First, we explored the relationship between neutral human-primate divergence and functional content, defined as the proportion of sites within a 100kb-window that overlapped with an exon or a phastCons region. We hypothesized that if natural selection contributes to the reduction of divergence at linked neutral sites, its effect would be more pronounced at regions with greater functional content [14]. This hypothesis predicts a negative correlation between functional content and neutral divergence. To test this, we divided the human genome into non-overlapping windows of 100kb and obtained putatively neutral divergence for each window as described above. We found a negative correlation between functional content and neutral divergence between pairs of closely related species (Spearman’s ρ_*human-chimp*_ = -0.235, *P* < 10^−16^, Spearman’s ρ_*human-orang*_ = -0.204, *P* < 10^−16^, Fig 2A and 2B, S2 Table).

We next examined the relationship between human-primate neutral divergence and broad-scale human recombination rate which we obtained from the deCODE genetic map [50]. While recombination has not been conserved throughout evolutionary history, the recombination rate at the broad-scale level (i.e. 100kb) was shown to be correlated between human and chimp [51,52]. We found a positive correlation between neutral human-primate divergence and human recombination rate (Spearman’s ρ_*human-chimp*_ = 0.234, *P* < 10^−16^, Spearman’s ρ_*human-orang*_ = 0.249, *P* < 10^−16^, Fig 2C and 2D, S3 Table), which indicates that neutral human-primate divergence is reduced in regions of low recombination rate. Additionally, when we stratified windows into those that were near genes and those that were far from genes based on the proportion of sites in each window that overlapped with a RefSeq transcript, we found that the correlation between divergence and recombination is stronger for windows with a higher overlap with RefSeq transcripts (S2 Fig). These observations indicate that neutral divergence is reduced at sites that are more tightly linked to those under the direct effect of selection, consistent with the hypothesis that natural selection indirectly reduces linked neutral divergence.

These two correlations are robust to the presence of multiple confounding factors. First, the correlations are robust to the choice of window size used for analysis as they persisted when using 50 kb windows (S2 and S3 Tables). Second, some features of the genome such as hypermutable CpG sites or GC content are known to correlate with genic content [10,12,13]. To test whether these features confounded the correlations found in our data, we repeated our analyses removing potential CpG sites by omitting sites preceding a G or following a C [15]. The correlations persisted after filtering out CpG sites (S2 and S3 Tables). We next computed partial correlations controlling for GC content. Similarly, we found that the correlations persisted (S2 and S3 Tables).

Biased gene conversion is an additional evolutionary force that has been shown to influence patterns of divergence [53,54]. In this process, double-strand breaks in the DNA in individuals heterozygous for AT/GC variants will be preferentially repaired with the GC allele, resulting in AT → GC substitutions occurring at a higher rate than GC → AT substitutions [54–56]. To control for the effects of biased gene conversion on this analysis, we filtered out sites that could be affected by removing any AT → GC substitutions genome-wide. The negative correlation between human-primate divergence and functional content did not change after controlling for biased gene conversion (S2 Table). Though the positive correlation between human-primate divergence and human recombination decreased after this filter (from 0.234 to 0.108), it still remained significant (S3 Table). Thus, the observed correlations are unlikely to be driven solely by choice of window size or mutational properties based on sequence composition. Because biased gene conversion appears to contribute to some of the correlation between divergence and recombination rate, subsequent analyses of this correlation use the divergence dataset filtered for biased gene conversion.

### Effects on putatively neutral divergence between humans and rodents

**Fig 3:**
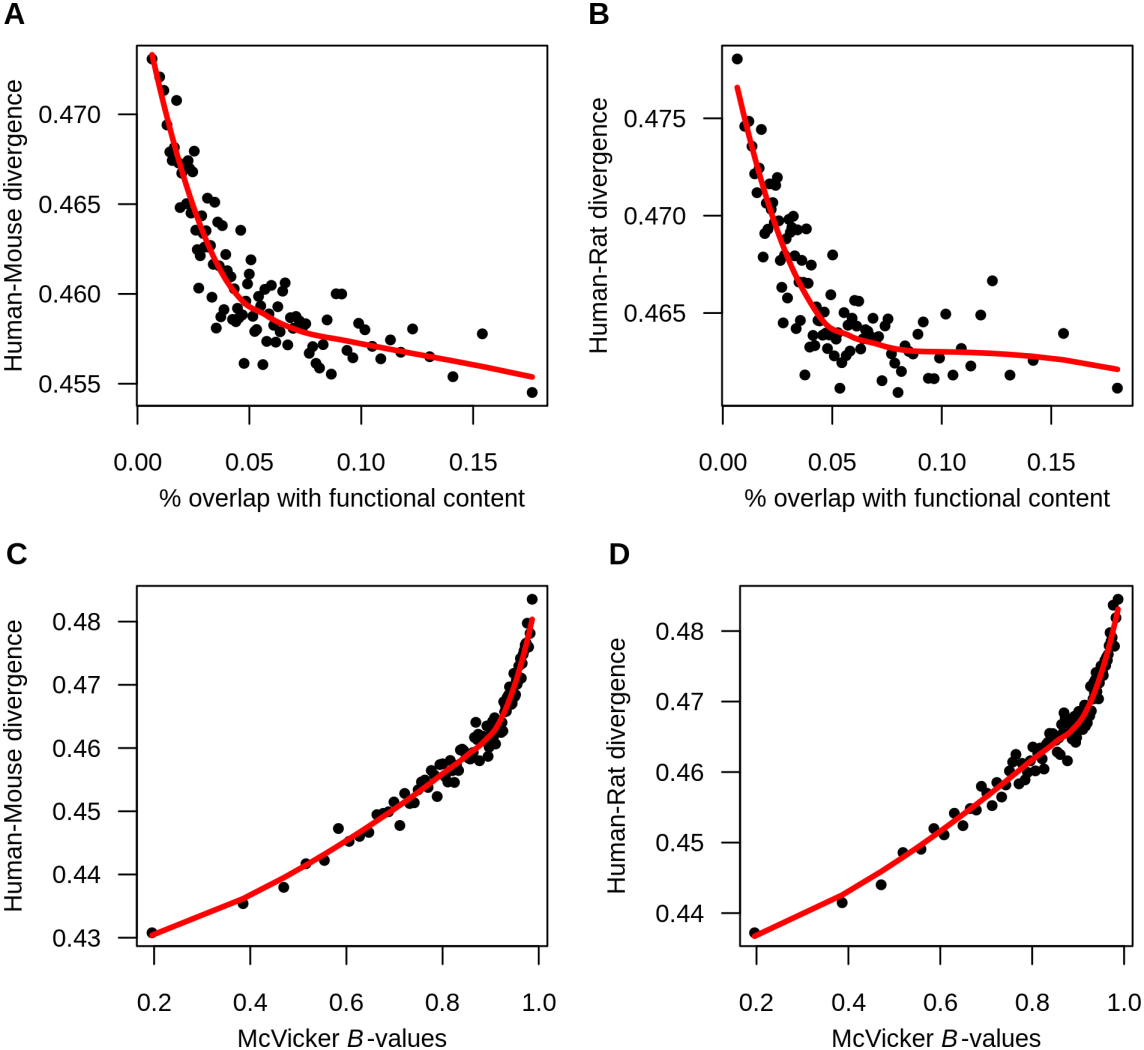
Human-rodent divergence is reduced at putatively neutral sites near selected sites. (A) Neutral human-mouse divergence is negatively correlated with functional content. (B) Neutral human-rat divergence is negatively correlated with functional content. (C) Neutral human-mouse divergence is positively correlated with McVicker’s *B*-values. (D) Neutral human-rat divergence is positively correlated with McVicker’s *B*-values. Each point represents the mean divergence and functional content (A and B) or *B*-values (C and D) in 1% of the 100kb windows binned by functional content or *B*-values. Red lines indicate the loess curves fit to divergence and functional content (A and B) and divergence and *B*-values (C and D). Note that the last bin containing less than 1% of the windows was omitted from the plot. While the graph presents binned data, the correlations reported in the text are from the unbinned data.

We next explored the evolutionary forces affecting divergence between more distantly related pairs of species, specifically human-mouse and human-rat. These species were predicted to have diverged approximately 75 million years ago [41] and, as such, current thinking would predict that natural selection would not affect linked neutral sites. Similar to what was seen for the closely related species, functional content is negatively correlated with neutral human-rodent divergence (Spearman’s ρ_*human-mouse*_ = -0.184, *P* < 10^−16^, Spearman’s ρ_*human-rat*_ = -0.149, *P* < 10^−16^, Fig 3A and 3B, S4 Table). This negative correlation persisted when using 50kb windows and also after accounting for the confounding factors of hypermutable CpG sites, GC content, and GC-biased gene conversion (S4 Table).

Since the broad-scale recombination rate at 100kb appears to have changed over the course of evolution of the species [57], we looked for other potential signatures of whether natural selection has affected linked neutral divergence. In particular, we examined the relationship between human-rodent divergence and the strength of background selection across the genome inferred from divergence within primates [15]. This strength of background selection is captured by the *B*-value, which represents the degree to which neutral variation at a given position is reduced by selection relative to neutral expectations. While McVicker et al. [15] concluded that divergence between primates was indeed reduced due to background selection, they did not consider human-mouse divergence in their analyses and did not model background selection within the human-dog ancestor. As such, there is no *a priori* reason why the *B*-values of McVicker et al. [15] should be related to human-mouse divergence.

Nevertheless, we found a positive correlation between human-rodent divergence and the *B-*values from McVicker et al. [15] (Spearman’s ρ_*human-mouse*_ = 0.445, *P* < 10^−16^, Fig 3C, S5 Table, Spearman’s ρ_*human-rat*_ = 0.402, *P* < 10^−16^, Fig 3D, S5 Table). The positive correlation between human-rodent divergence and *B*-values remained significant even after accounting for the confounding factors of CpG sites, GC content, and GC-biased gene conversion. Similarly, these correlations remained when using 50kb windows (S5 Table). Taken together, the empirical correlations are consistent with the hypothesis that natural selection has contributed to reducing neutral divergence at linked sites even between species with a long split time such as human and mouse.

### Models incorporating background selection in the ancestral population can generate the empirical correlations

To test whether a model including background selection in the ancestral population can explain the empirical observations regarding neutral human-primate divergence and neutral human-rodent divergence, we used a coalescent simulation approach. To a first approximation, the effect of background selection in a sample size of two chromosomes can be accounted for by scaling the ancestral population size by the strength of background selection [4,5,7,15,58–62]. Thus, we modeled the effect of background selection as a reduction in the ancestral population size using the *B*-values estimated in McVicker et al. [15]. Briefly, we first used *ms* [63] to generate genetic variation in the ancestral population where the ancestral population has size *N*_*a*_*B*. Then we simulated mutations that accumulated since the split between two species using a Poisson process. The total divergence was the sum of the mutations in the ancestral population and mutations accumulated since the split (see Methods). We modeled mutation rate variation by drawing a mutation rate for each window from a gamma distribution. We chose values for the parameters of the gamma distribution as well as the ancestral population size (*N*_*a*_) such that the mean and standard deviation of the simulated divergence across the genome and the correlation coefficients between divergence and other functional properties were similar to those seen empirically (S3 Fig, S6 and S7 Tables, Methods).

**Fig 4:**
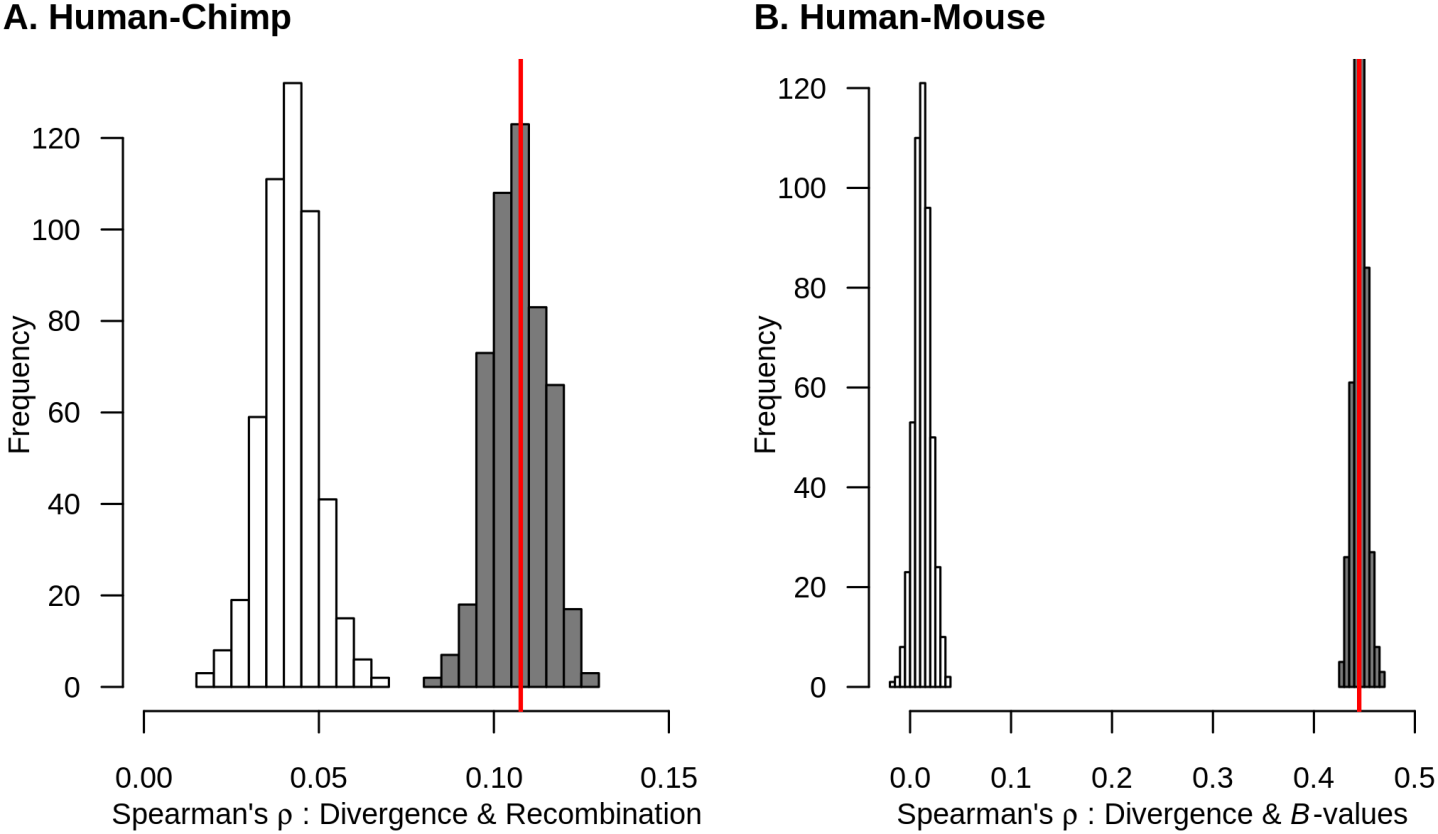
Models incorporating background selection can generate patterns of neutral divergence that recapitulate the empirical correlations. (A) Models of background selection predict a positive correlation between neutral human-chimp divergence and human recombination. Because our model does not include biased gene conversion, the empirical correlation was calculated omitting AT to GC sequence differences. (B) Models of background selection predict a positive correlation between neutral human-mouse divergence and McVicker’s *B*-values. White histogram denotes 500 simulations not including background selection. Gray histogram denotes 500 simulations incorporating background selection (see text). Red line represents the correlation computed from empirical data. Thus, plausible levels of background selection can match the observed correlations while neutral simulations cannot.

We first examined which models could generate the observed correlation between recombination and human-chimp divergence. Here we use the value of Spearman’s ρ estimated from the data after filtering out sites that could be affected by biased gene conversion (ρ = 0.108). When considering a model without background selection (i.e. *B* = 1 for all windows), the average value of Spearman’s ρ between human-chimp divergence and recombination rate was 0.042, and none of the 500 simulation replicates approached the value of Spearman’s ρ seen empirically (Fig 4A**, white histogram**). On the other hand, when modeling background selection using the McVicker *B*-values, the average Spearman’s ρ was 0.107 which was comparable to the Spearman’s ρ computed from empirical human-chimp divergence with human recombination after accounting for biased gene conversion (Fig 4A**, gray histogram**).

We then tested whether a model incorporating background selection could generate a positive correlation between neutral human-rodent divergence and *B*-values as observed empirically. We modified our simulation approach to account for the difference in generation time between human and mouse (see Methods). When considering models without background selection (i.e. *B* = 1 for all windows), the average value of Spearman’s ρ was 0.012, and none of the 500 simulation replicates approached the value of Spearman’s ρ seen empirically (Fig 4B**, white histogram**). However, when modeling background selection using the McVicker *B*-values, the average Spearman’s ρ was 0.446 which was comparable to the Spearman’s ρ computed from empirical human-mouse divergence and McVicker’s *B*-values (Fig 4B**, gray histogram**).

In sum, our results suggest that for a given set of parameters, a model with background selection in the ancestral population can generate the correlations observed in the empirical data (i.e. a positive correlation between neutral human-primate divergence and human recombination and a positive correlation between neutral human-rodent divergence and *B*-values) whereas neutral coalescent models cannot.

### Intuition for why background selection is a plausible explanation for the empirical correlations

**Fig 5:**
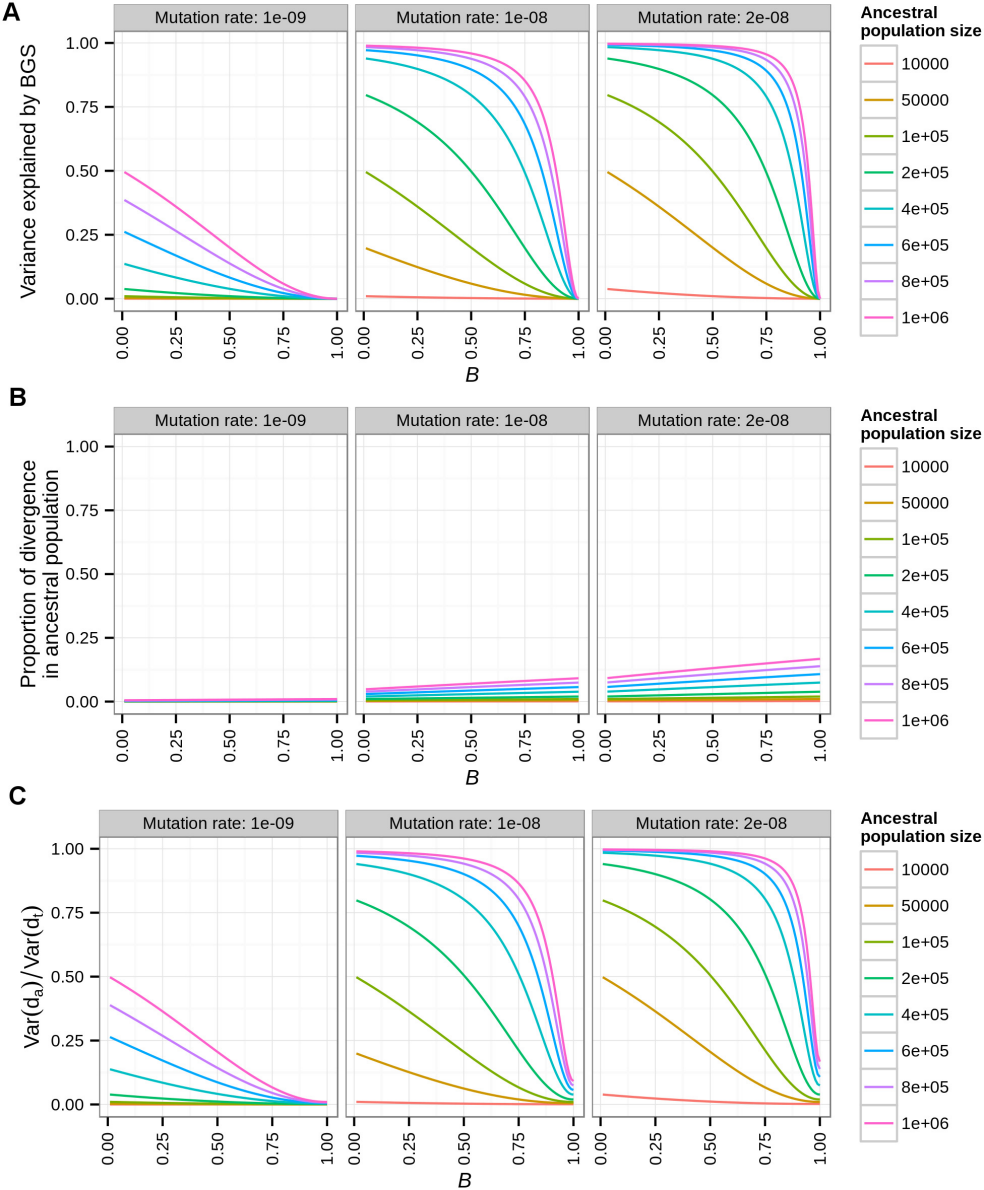
A two-locus model for the effect of background selection on divergence. (A) The variance in divergence between two loci explained by background selection (BGS) as a function of the strength of background selection at the second locus (*B*_*2*_). (B) The expected proportion of divergence due to polymorphism in the ancestral population as a function of *B*_*2*_. (C) The variance in divergence between the two loci explained by polymorphism in the ancestral population as a function of *B*_*2*_. Different columns denote different mutation rates. Colored lines denote different ancestral population sizes (*N*_*a*_). Note that the variance in divergence attributable to background selection is greater than the expected proportion of divergence contributed by ancestral polymorphism.

Current thinking argues that natural selection affecting linked neutral sites is not a plausible explanation for the reduction in neutral divergence between pairs of species with a long split time such as human-mouse or human-rat. Here, we outline a theoretical analysis of a simple two-locus model to gain intuition about how the mutation rate (*μ*), strength of background selection (*B*), and ancestral population size (*N*_*a*_) affect the degree to which background selection can affect divergence (Fig 5A).

If background selection has any effect on the variation in neutral divergence across the genome, this can only be due to its effect on divergence in the ancestral population, since deleterious mutations do not affect the fixation rate at linked neutral sites [18]. Recombination in the ancestral population results in a distribution of coalescent times within each locus, with an average coalescent time of $\bar{t}$. We assumed that the recombination rate within each locus is large enough, such that there is no variation in $\bar{t}$ for a fixed value of *B*, i.e. $\text{Var}\left[\bar{t}|\text{B}\right]\approx 0$. This is a reasonable assumption as long as the window size and recombination rate are not too small. Recombination events cause the sequence to be broken into independent segments, such that for a total *ρ* > 10 (where *ρ* denotes the population-scaled recombination rate, 4*N*_*e*_*r*) the variance in $\bar{t}$ approaches zero [64]. For an average 100kb window in the human genome (*r* = 10^−8^/bp, N_e_ = 10,000), *ρ* is 40 and thus this assumption holds true. Any difference in $\bar{t}$ between loci is then only attributable to differences in background selection: $\text{E}\left[\bar{t}|B\right]=2N_{a}B$. Further, variation in ancestral (*d*_*a*_) and total (*d*_*t*_) divergence results from a Poisson distributed number of mutations added to the genealogy, such that *Var*[*d*_*a*_|*B*] = *E*[*d*_*a*_|*B*] = 4*N*_*a*_*BμL* and *Var*[*d*_*t*_|*B*] = *E*[*d*_*t*_|*B*] = *E*[*d*_*a*_|*B*]+2*t*_*split*_*μL* where *L* is the sequence length of a locus. The law of total variance can be used to compute the variance in total divergence across loci with varying levels of background selection:

$$Var\left[d_{t}\right]=Var_{B}\left[E\left[d_{t}|B\right]\right]+E_{B}[Var[d_{t}|B]]$$

Thus, variance in total divergence can be decomposed into variance due to background selection and variance due to the mutational process. For simplicity, the first locus experiences no background selection (*B*_1_ = 1), and the second locus experiences some fixed amount of background selection (0 ≤ *B*_2_ ≤ 1). Under this model, we computed the variance due to background selection as:

$$Var_{B}\left[E\left[d_{t}|B\right]\right]={\left(\left(E\left[d_{t}\left|B=1]\;–\;E[d_{t}\right|B=B_{2}\right]\right)/2\right)}^{2}.$$

We then computed the variance due to the mutational process as:

$$E_{B}\left[Var\left[d_{t}|B\right]\right]=\left(Var\left[d_{t}|B=1\right]+Var\left[d_{t}|B=B_{2}\right]\right)/2.$$

We assumed an old split time, such that the divergence that accumulated from present time to population split is similar to the human-mouse divergence (40%). Both loci have a sequence length (*L*) of 100kb. Our theoretical analysis of variance approach shows that with this old split time and assuming a low mutation rate of 1 x 10^−9^/bp, more than 20% of the variation in the divergence can be explained by background selection in the ancestral population with the following conditions: ancestral population size > 600,000 and *B* < 0.2 (Fig 5A**, panel 1, blue, purple, and pink lines**). Note that under these conditions, the proportion of divergence that accumulated in the ancestral population can be as low as 0.3% (Fig 5B**, panel 1**). However, the proportion of the variance in divergence that is attributable to the ancestral population is larger than 20% (Fig 5C**, panel 1**), mainly due to background selection leading to differences in $\bar{t}$ between loci. With a larger mutation rate (2 x 10^−8^/bp), background selection results in a stronger effect on variation in divergence even when ancestral population size is relatively small (>50,000; Fig 5A**, yellow line**). When assuming a moderately large population size of 200,000, and a moderate strength of background selection (*B* = 0.75), then as much as 50% of variance in divergence can be explained by background selection (Fig 5C, **light green line**). Nonetheless, the proportion of divergence that accumulated in the ancestral population in this case is still only 3.4%. Collectively, even for old split times, where the vast majority of divergence accumulated after the population split, with certain assumptions about the ancestral population size, mutation rate, and strength of background selection, the variance in the divergence could be explained by background selection.

### Coalescent simulations predict background selection can reduce neutral divergence between species with long split times

Because the theoretical model described above ignores regions of low recombination and only considers one pair of loci at a time, we used coalescent simulations (similar to what we outlined above) to examine whether background selection could generate the positive correlation between estimates of background selection in primates and divergence between distantly related species using more realistic models. Since we were not particularly concerned with any specific species, we simplified these simulations by setting the mutation rate to 2.5 x 10^−8^/bp.

We found that across all population sizes and split times examined, background selection generated a positive correlation between recombination and divergence as well as a positive correlation between divergence and *B*-values, even for pairs of species that split up to 100*N* generations ago (S4 Fig**, black lines and dashed lines**). This correlation remained strong even when the proportion of the divergence due to ancestral polymorphism was small. For example, for a pair of populations with t_split_ = 100*N* generations and an ancestral population of size 50,000, only 1.53% of the divergent sites are due to ancestral polymorphism (S4 Fig**, red lines**). However, this model predicts a correlation of 0.211 between recombination and divergence and a correlation of 0.377 between recombination and *B*-values. Although ancestral polymorphism only contributes in a small way to the total divergence, the variance in the amount of ancestral polymorphism across the windows accounts for nearly 60% of the variance in divergence across different windows (S5 Fig**, black lines**). In general, the correlations decreased as both the split time increased and the size of the ancestral population decreased (S5 Fig). This behavior is expected as the contribution of the variance in levels of ancestral polymorphism to the variance in divergence decreases with increasing split time and decreasing ancestral population size (S5 Fig).

### Examining the direct effects of natural selection on observed correlations

While we have shown under a variety of models that natural selection can affect putatively neutral divergence and generate the correlations that we observe empirically, other selective scenarios could explain these patterns. An alternative explanation for the empirical correlations reported in Figs 2 and 3 is that the filtering criteria we used to obtain neutral sites did not effectively remove all non-neutral sites. Therefore, the observed correlations could be due to the direct effects of purifying selection reducing genetic divergence. As sites under purifying selection may be located close to conserved functional elements and could conceivably result in low *B*-values, this is a potentially plausible explanation for our findings. As our current filters removed the 10% of the genome that was most likely under the direct effect of selection based upon the top 10% of GERP scores, we reasoned that additional sites under purifying selection would have elevated GERP scores relative to neutrality.

**Fig 6:**
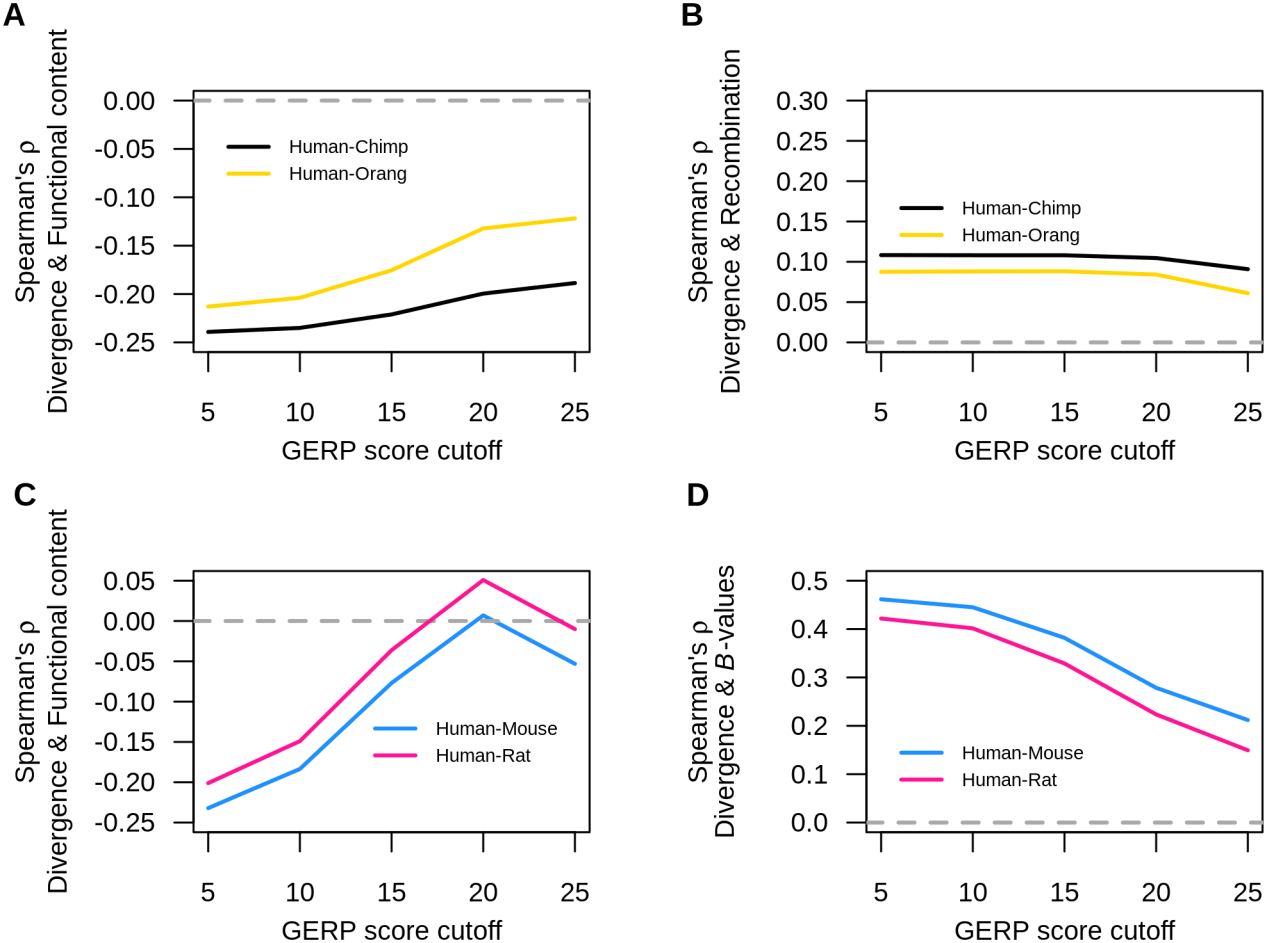
Relationship between divergence and functional content, human recombination, and McVicker’s *B*-values as a function of GERP score cutoff. (A) Human-primate divergence versus functional content. (B) Human-primate divergence versus human recombination rate. (C) Human-rodent divergence versus functional content. (D) Human-rodent divergence versus McVicker’s *B*-values.

To test this hypothesis, we repeated our correlation analyses by first obtaining the neutral human-primate divergence and neutral human-rodent divergence using different GERP score cutoffs (i.e. 5% to 25%). When examining human and primate pairs, the correlation between neutral human-primate divergence and functional content decreased as a function of increasing GERP cutoff score (Fig 6A). Nevertheless, the negative correlation between neutral human-primate divergence and functional content remained significant even after removing any site whose GERP score fell within the top 25% of the distribution (Spearman’s ρ_*human-chimp*_ = -0.189, *P* < 10^−16^, Spearman’s ρ_*human-orang*_ = -0.122, *P* < 10^−16^, S6A and S6B Fig). On the other hand, the relationship between neutral human-primate divergence and human recombination rate were not affected by varying GERP score cutoffs (Fig 6B, S6C and S6D Fig).

When examining human and rodent pairs, we found that the negative correlation between human-rodent divergence and functional content decreased as a function of increasing GERP score cutoff. Further, the relationship became nonsignificant when filtering any site whose GERP score fell within the top 15th percentile (Fig 6C, S7A and S7B Fig). The positive correlation between neutral human-rodent divergence and McVicker’s *B* values decreased as a function of increasing GERP score cutoff, but remained significantly positive even after removing any sites whose GERP score fell within the top 25th percentile (Fig 6D, S7C and S7D Fig). Still, this latter pattern indicates that the direct effects of natural selection are unlikely to explain our findings, unless the selected sites are not in the upper 25% of the GERP score distribution.

To test whether background selection could explain these correlations when removing the 25% of the genome with the most conserved GERP scores, we used our coalescent simulation framework. These simulations match the empirical distribution of divergence across the genome (S8 Fig, S7 Table) and use the parameters given in S6 Table. For human-chimp divergence, none of the 500 neutral coalescent simulations resulted in a Spearman’s ρ between divergence and human recombination rate as large as observed empirically after filtering sites affected by biased gene conversion (S9A Fig**, white histogram**). On the other hand, simulations including background selection in the ancestral population generated a Spearman’s ρ between divergence and human recombination rate similar to what was observed empirically after filtering sites affected by biased gene conversion (S9A Fig**, gray histogram**). Similarly, for human-mouse divergence, while none of the 500 coalescent simulations using the neutral model could generate a Spearman’s ρ between divergence and McVicker’s *B*-values as large as the empirical correlation, models including background selection in the ancestral population could generate this correlation (S9B Fig).

## Discussion

Here we have examined patterns of divergence between pairs of species with various degrees of divergence. We document several signatures that are consistent with the action of natural selection reducing divergence at linked neutral sites. First, for all pairs of species considered, we find that neutral divergence is lowest in regions of the genome with the greatest functional content (Figs 2 and 3). This pattern may be expected if more selection occurs in regions of the genome with greater functional content. Second, human-primate neutral divergence strongly correlates with human recombination rate and the correlation persists after accounting for hypermutable CpG sites, GC content, and biased gene conversion. Regions of low recombination show lower levels of divergence, which is consistent with selection having a greater effect on linked neutral sites in regions of low recombination. The correlation between human-primate divergence and human recombination is higher in regions with greater overlap with RefSeq transcripts, indicative of a greater reduction in neutral divergence in regions near genes as opposed to far from genes (S2 Fig). Third, human-rodent neutral divergence strongly correlates with the strength of background selection estimated for primates. These correlations persist after accounting for CpG sites, GC content, and biased gene conversion. Importantly, coalescent simulations including background selection can generate several of these correlations. However, neutral coalescent models without background selection do not.

One interesting observation made was that while most of our correlation analyses were robust to the confounding effect of biased gene conversion, the correlation between human-primate neutral divergence and recombination rate was affected significantly by biased gene conversion. This suggests that while some of the correlation between recombination and divergence can be driven by biased gene conversion, it cannot explain the entire correlation. This result also argues that when testing for a correlation between divergence and recombination, the effect of biased gene conversion should be taken into account.

While we found that models incorporating background selection predict correlations comparable to the empirical data, in principle, several other evolutionary processes may be able to generate these patterns. First, selective sweeps in the ancestral population could reduce divergence just like background selection. Given that we are unlikely to be able to survey patterns of polymorphism in the human-mouse ancestor in more than two lineages, it will be difficult or nearly impossible to distinguish between these two types of selection at linked neutral sites. Thus, one should interpret our use of *B*-values as reflecting a reduction in divergence due to the combined effects of both background selection and selective sweeps, as suggested in McVicker et al. [15].

A second possibility is that the negative correlation between divergence and functional content as well as the positive correlation between divergence and *B*-values could be driven by variation in mutation rate across the genome. Indeed, McVicker et al. [15] attributed a positive correlation between *B*-values and human-dog divergence to the effects of variable mutation rates. However, for this mechanism to explain our results, it would require that mutation rates would have to be lower closer to genes and in regions of the genome thought to experience more background selection (i.e. in regions with lower *B*-values). There is some limited evidence of this effect in Arabidopsis where mutation rates are higher in regions of the genome with greater heterozygosity [65]. However, the extent to which these results apply to mammalian genomes remains unclear. Further, other studies in humans do not support the view that mutation rates are systematically lower in regions of the genome more subjected to selection. Recent estimates of the *de novo* mutation rate have not found any evidence of a reduction close to genes [32]. Further, Palamara et al. [66] found that their estimates of the mutation rate do not differ as a function of *B*-values. Variation in mutation rate across the genome, while inflating the variance in divergence across the genome, would not be predicted to generate correlations between *B*-values and divergence as well as the correlation between functional content and divergence. Thus, we can rule it out as the sole explanation for the empirical patterns seen in our study.

Further, mutagenic recombination is unlikely to explain the empirical patterns in our study because the correlation between divergence and functional content does not depend on recombination rate. The negative correlation between divergence and functional content remained strong when controlling for variation in recombination rates (S2 Table), suggesting our results are unlikely to be driven by mutagenic recombination. Nevertheless, our results do not rule out the possibility of mutagenic recombination and this topic certainly warrants further investigation.

Another possibility is that the reduction in neutral divergence near genes and in regions with lower *B*-values could be due to the direct effects of purifying selection removing variation from the population. Current evidence from a variety of comparative genomic studies suggests <10% of the genome is under purifying selection [39,41–49]. We attempted to mitigate the direct effects of purifying selection by employing a conservative set of filters in order to obtain putatively neutral sites. When removing the 10% of the genome that is most conserved, using a variety of conservation metrics, the correlations persisted, suggesting they were not driven by the direct effects of selection. However, when we removed the top 15% of sites with the most conserved GERP score, the correlation between human-rodent divergence and functional content disappeared. This finding suggests that either the GERP scores themselves are affected by background selection, or, instead, that this correlation is driven, in part, by the direct effects of purifying selection. However, in order for direct purifying selection to explain the correlation, either more of the genome (at least 15%) would have to be under selection than suggested by current estimates [39,41–49] or many of the sites in the top 15% most conserved GERP scores would have to be neutrally evolving. Additionally, the negative correlation between human-chimp divergence and functional content, the positive correlation between human-chimp divergence and recombination rate, and the positive correlation between human-mouse divergence and *B*-values, remained even after removing the 25% of the genome that is most conserved (Fig 6). This implies that even such a large amount of functional sites under selection cannot explain all of our results. Finally, an additional line of evidence suggesting that the putatively neutral sites close to genes are not subjected to the direct effects of purifying selection stems from the fact that they show similar levels of neutral divergence to four-fold degenerate sties (S1 Fig, S1 Table). Thus, our putatively neutral noncoding sites have levels of divergence comparable to those seen for sites solely subjected to background selection.

Additionally, our filters rely on functional annotations and conservation to remove functionally important sites directly under the effects of selection. It is formally possible that the direct effects of selection could generate the correlations seen in our study if there are sites under selection that were invisible to the conservation-based filters used in our study. This could occur if there are recently derived, lineage-specific functional elements under selection that cannot be picked up by conservation metrics, or if there are sequences subject to purifying selection in the ancestral population but subsequently became neutral and therefore were not conserved. While we cannot exclude such a scenario, current population genetic evidence provides, at most, limited support for such an explanation [44,46,47,67].

One limitation in this study is that we made many assumptions regarding the parameters used in the simulations such as the ancestral population size, generation times, and mutation rates over the last 5–7 million years between human and chimp and 75 million years between human and mouse. There is much uncertainty surrounding all of these parameters [41,68–72]. Overall, we used a set of parameters in which the simulated divergence dataset from the coalescent simulations matched closely with the mean and standard deviation of the empirical divergence dataset. This allowed us to assess whether a simple neutral model could result in the correlations as large as observed empirically or whether a model with background selection needed to be invoked. We utilized the coalescent simulations as a proof of concept and therefore, the parameters we used in these sets of simulations should not be taken as estimates of the true values. Estimation of these parameters (ancestral population size, mutation rate, split time, etc.) is beyond the scope of this study and certainly warrants further in-depth investigation.

Other studies have argued that selection will not affect linked neutral divergence between distantly related species because the genealogy in the ancestral population only comprises a small proportion of the total genealogy between one chromosome from each of the two species [9,18,27]. This means that ancestral polymorphism will only account for a small proportion of the total divergence between distantly related species. It was thought that the signature of selection reducing the genealogy in the ancestral population would be diluted by the mutations that occurred since the split. As such, there would be no detectable signature of selection. Our theoretical results and simulations show the proportion of ancestral polymorphism actually is a poor predictor of the correlation between divergence and recombination as well as between divergence and *B*-values. For example, consider a pair of species that split *N* generations ago with an ancestral population size of 25,000. In this model, 40% of the divergence is attributable to ancestral polymorphism (S4A Fig). Now consider a second pair of species that split 100*N* generations ago where *N*_*a*_ = 200,000. Here <5% of the divergence is due to ancestral polymorphism (S4D Fig). Previous intuition suggests the effect of background selection would be stronger in the first pair of species because they split more recently and ancestral polymorphism makes a greater contribution to divergence. However, our simulations show the exact opposite pattern (S4A and S4D Fig). The correlation between *B*-values and divergence is higher in the model with the more ancient split (Spearman’s ρ = 0.610) than the one with the more recent split (Spearman’s ρ = 0.452). Similar results are seen for the correlation between recombination rate and divergence. The reason for this discrepancy is that the main driver of these correlations is not the average amount of ancestral polymorphism, but rather the contribution to the variance in divergence due to the variance in ancestral polymorphism. Even when ancestral polymorphism makes only a small contribution to the overall average divergence, a substantial amount of the variance in total divergence across the genome can still be explained by variance in ancestral polymorphism, particularly if the ancestral population size is large. Our theoretical results suggest that the variance in the amount of background selection in different regions of the genome can account for a lot of the variance in total divergence, even for species that split long ago. In sum, our theoretical results and simulations suggest that previous intuition has understated the importance of even small amounts of ancestral polymorphism on the variability of genome-wide patterns of divergence between species.

Our results have important implications for understanding patterns of genetic variation and divergence across genomes. First, our findings add to the growing literature suggesting the importance of background selection at shaping genome-wide patterns of variability across species [1,7,13,15,16,60,62,73–80]. Our new contribution to this literature is demonstrating that natural selection can affect neutral divergence, even between distantly related species. Second, our work suggests that estimators of mutational properties that rely on contrasting patterns of divergence across different parts of the genome that may be differentially affected by background selection may yield biased results. This effect has been studied within primates in greater detail in recent work [81]. Third, the fact that we detect evidence of background selection between distantly related species suggests that there is still some information about the distribution of coalescent genealogies across the genome. This distribution of coalescent genealogies can be exploited to obtain more reliable estimates regarding the human-mouse ancestral population size. While several methods exist to estimate ancestral demographic parameters from divergence [82–86], we suggest that these methods may be applicable for very distantly related species. Our finding that background selection can increase the variance in coalescent times across the genome suggests these methods as well as other statistical methods which seek to infer demographic history from the distribution of coalescent times across the genome, such as the PSMC approach [87], should account for the increased variance in coalescent times across the genome due to background selection. Not accounting for background selection could result in inferring spurious demographic events to account for the additional variance in coalescent times across the genome as has recently been suggested for positive selection [88]. Lastly, our results suggest a need for caution when using patterns of divergence to calibrate neutral mutation rates. Some of the variation in divergence across the genome may be due to varying coalescent times, further accentuated by selection, rather than differing mutation rates [20,89]. Future work could explore the extent to which selection at linked neutral sites can explain the discrepancies between different types of estimates of mutation rates [33,34].

## Methods

### Data sets

We obtained the pairwise (.axt) alignments between human/chimpanzee (hg18/panTro2), human/orang (hg18/ponAbe2), human/mouse (hg18/mm9), human/rat (hg18/rn4), and human/zebrafish (hg18/danRer15) from the UCSC genome browser [90]. These alignments are the net of the best human chained alignments for each region of the genome [91]. For quality control, we excluded sites that (1) were missing in either of the species in the alignment, (2) were located within 10Mbp from the starting or ending position of a centromere, (3) were located within 10Mbp from the ending position of a telomere, (4) were located in repetitive elements.

We obtained the coordinate positions for the exons, RefSeq transcripts, and different phastCons measures calculated from different phylogenetic scopes [82] from the UCSC table browser [92] with the following specifications:

1. Exons: clade: Mammal, genome: Human, assembly: Mar. 2006 (NCBI36/hg18), group: Genes and Gene predictions, track: UCSC Genes, table: knownGene.
2. RefSeq transcripts: clade: Mammal, genome: Human, assembly: Mar. 2006 (NCBI36/hg18), group: Genes and Gene predictions, track: RefSeq Genes, table: refGene.
3. phastCons Vertebrates: clade: Mammal, genome: Human, assembly: Mar. 2006 (NCBI36/hg18), group: Comparative Genomics, track: Conservation, table: Vertebrate El (phastConsElements44way).
4. phastCons Primates: clade: Mammal, genome: Human, assembly: Mar. 2006 (NCBI36/hg18), group: Comparative Genomics, track: Conservation, table: Primate El (phastConsElements44wayPrimates).
5. phastCons Mammals: clade: Mammal, genome: Human, assembly: Mar. 2006 (NCBI36/hg18), group: Comparative Genomics, track: Conservation, table: Mammal El (phastConsElements44wayPlacental).

GERP scores were downloaded for hg18 from http://mendel.stanford.edu/SidowLab/downloads/gerp/. We used RS scores (range from -11.6 to 5.82) to obtain the conserved sites to remove. S8 Table summarizes the cutoffs we used.

For each window, we computed the recombination rate using the high resolution pedigree-based genetic map assembled by deCODE [50]. The *B*-value for each window was obtained from McVicker et al. [15]. Four-fold divergence was calculated by counting the number of between species differences that overlapped four-fold sites, divided by the total number of four-fold sites within each window. Functional annotation was done following Lohmueller et al. [13]. Briefly, we translated the Consensus Coding Sequence (CCDS) genes from the UCSC Genome Browser into proteins and determined which nucleotide changes did not alter the encoded amino acid. If transcripts overlapped, we retained the longest one.

### Correlation analyses

To calculate the divergence between each pair of species, we divided the human genome into 100kb non-overlapping windows. For each window, we computed the total number of sites that passed the filtering criteria which resulted in the total number of neutral sites in each 100kb window. To reduce variation, we only considered windows in which the total number of eligible sites was greater than 10,000 (for analyses using 50kb as window size, we only considered windows in which the total number of eligible sites was greater than 5,000). Then we computed the divergence by tabulating the number of sites that are different between the two species being compared. To account for multiple mutational hits for the distantly related species pairs (human-mouse and human-rat), we applied the Kimura two-parameter model [93].

To compute Spearman's ρ, we used the *cor* function in R. We used the *pcor* function to calculate partial correlation [94].

### Controlling for confounding factors

To filter out possible hypermuteable CpG sites, we excluded sites that were preceded by a C or were followed by a G in hg18 [15]. To control for the effects of biased gene conversion, we removed all AT→GC substitutions across the genome.

### Coalescent simulations

We modeled background selection as a simple reduction in effective population size in the ancestral population [4,5,7,15,58–62]. This was done by scaling the ancestral population size *N*_*a*_, by the *B-*values. We used the *B*-values from McVicker et al. [15]. Each simulation replicate consisted of two parts. The first part modeled genetic variation in the ancestral population, and included the effects of background selection. For each window *i*, we simulated an ancestral recombination graph (ARG) with a population-scaled recombination rate 4*N*_*a*_*B*_*i*_*r*_*i*_, where *N*_*a*_ is the ancestral population size, *B*_*i*_ is the strength of background selection affecting window *i*, and *r*_*i*_ is the recombination rate for window *i*. Mutations were added to the genealogy assuming a population-scaled mutation rate *θ* = 4*N*_*a*_*B*_*i*_*μ*_*a*,*i*_*L*_*i*_, where *μ*_*a*,*i*_ is the ancestral per-base pair mutation rate for window *i* and *L*_*i*_ is the number of successfully aligned neutral bases in window *i*. Simulations were done using the program *ms* [63]. Note, we included recombination in the ancestral population because it affects the variance in coalescent times across windows and this variance in coalescent times will in turn affect the variance in levels of divergence, which will ultimately affect the strength of the correlation between divergence and recombination. Thus, we aimed to capture this variance as accurately as possible. This part of the simulation generated the amount of divergence due to ancestral polymorphism, which we call *d*_*a*_.

We then added the mutations that arose since (i.e. more recently than) the split. The divergence from the present time to the split time follows a Poisson distribution, where the rate parameter equals the expected divergence between two populations. For each window of the genome, *d*_*s*_ was simulated using the *rpois* function in R. Finally, the total divergence within a window is the sum of divergence generated in the ancestral population (*d*_*a*_) and the divergence generated since the two species split (*d*_*s*_).

For human chimp divergence (Fig 4A, S9A Fig), *d*_*s*_ = 2*t*_*split*_*μL* where *d*_*s*_ is the expected divergence from the present time to the split time in the divergence model, *t*_*split*_ is the split time, *μ* is the mutation rate, and *L* is the length of each sequence. When computing both *d*_*a*_ and *d*_*s*_ for human-chimp divergence in Fig 4A, we drew *μ* from a gamma distribution with shape = 16.82 and scale 1.7 X 10^−10^ (S6 Table). In S9A Fig, we drew *μ* from a gamma distribution with shape = 15.68 and scale 1.8 X 10^−10^ (S6 Table). These parameters were chosen to match the observed mean and standard deviation of the distribution of human-chimp divergence (after removing all AT to GC differences as such changes could be due to biased gene conversion) as well as the observed correlation coefficient between divergence and recombination rate (S3A, S3B, S8A and S8B Figs). The split times and ancestral population sizes are roughly comparable to previous estimates from genetic data [84,85,95,96].

Due to the differences in generation times and mutation rates between the human and mouse lineages, we modified our approach for these simulations (Fig 4B, S9B and S10 Figs). First, here *d*_*s*_ *=* (*t*_*mouse*_ *μ*_*mouse*_+ *t*_*human*_*μ*_*human*_)*L*, where *t*_*mouse*_ is the number of generations on the lineage leading to the mouse from *t*_*split*_ till the present day, *t*_*human*_ is the number of generations on the lineage leading to human experienced from *t*_*split*_ till the present day, *μ*_*mouse*_ is the mutation rate along the mouse lineage, and *μ*_*human*_ is the mutation rate along the human lineage. There is much uncertainty surrounding these parameters. However, the following values are broadly consistent with what has been reported previously and match the observed mean and standard deviation of human-mouse divergence (S3C, S3D, S8C and S8D Figs, S7 Table). First, we assumed *t*_*split*_ = 75 million years ago. We then assumed mice have 1 generation per year, giving *t*_*mouse*_ = 75 x 10^6^ generations. We assumed humans have 25 years per generation, making *t*_*human*_ = 3 x 10^6^. We then set *μ*_*mouse*_ = 3.8 x 10^−9^ per generation and *μ*_*human*_ = 3.75 x 10^−8^ per generation (S10 Fig). These estimates are broadly consistent with previous reports and allow for approximately twice as much divergence on the mouse lineage as compared to the human lineage [41].

For the simulations in Fig 4B, we assumed that *μ*_*a*_ was equal to 2 x 10^−8^ per generation, which is the average of *μ*_*human*_ and *μ*_*mouse*_. We accounted for variation in mutation rates across different regions of the genome by drawing *μ*_*a*_ from a gamma distribution [97]. We kept the ratio of *μ*_*a*_ to *μ*_*mouse*_ constant across all windows of the genome. For example, *μ*_*a*_ / *μ*_*mouse*_ = 5.26. Then if *μ*_*a*,i_ is the rate for the *i*^th^ region drawn from the gamma distribution, we set *μ*_*mouse*,*i*_ equal to *μ*_*a*,i_ / 5.26. A similar procedure was used to find *μ*_*human*,*i*_. Note that for the simulations in S9B Fig, we used the average mutation rate of 2.7 X 10^−8^, but we kept the ratio of *μ*_*a*_ to *μ*_*mouse*_ and the ratio of *μ*_*a*_ to *μ*_*mouse*_ to be the same as the simulations in Fig 4B. Increasing the variance in the mutation rate across regions increased the variance in divergence across windows of the genome and decreased the correlation between divergence and the *B*-values. We then examined different values of *N*_*a*_ and parameters of the gamma distribution that matched the observed mean and standard deviation of the distribution of human-mouse divergence as well as the observed correlation coefficient between divergence and *B*-values. The ancestral population size, shape, and scale parameters of the gamma distribution used for the simulations in Fig 4B and S9B Fig are reported in S6 Table. The simulated human-mouse divergence using these parameters matched closely with the empirical human-mouse divergence (S3C, S3D, S8C and S8D Figs, S7 Table).

## Supporting Information

S1 Fig Four-fold degenerate sites show similar levels of divergence as our putatively neutral noncoding sites. Each point represents the divergence within a 100kb window. (A) Human-chimpanzee, (B) Human-orangutan, (C) Human-mouse, and (D) Human-rat.
(PDF)

S2 Fig The correlation between human recombination rate and neutral divergence is stronger near genes. Correlation (Spearman’s ρ) between neutral divergence and human recombination as a function of the amount of overlap with a RefSeq transcript. Black line denotes the correlations between human-chimpanzee neutral divergence and human recombination rate. Yellow line denotes the correlations between human-orangutan neutral divergence and human recombination rate.
(PDF)

S3 Fig Observed and modeled genome-wide distributions of human-chimp divergence and human-mouse divergence in 100 kb windows. Gray lines denote 500 simulated genome-wide distributions of divergence. Red line denotes the observed distribution of neutral divergence. Note, the distribution of simulated divergence is comparable to that from empirical data. (A) Simulated human-chimp divergence without the effects of background selection (BGS). (B) Simulated human-chimp divergence with the effects of background selection. (C) Simulated human-mouse divergence without the effects of background selection. (D) Simulated human-mouse divergence with the effects of background selection. We filtered all AT→GC changes between the human and chimp sequences as they could be affected by biased gene conversion. Thus, the distribution of human-chimp divergence shown here is lower than the overall divergence.
(PDF)

S4 Fig Background selection is predicted to affect neutral divergence across a range of split times and ancestral population sizes. Solid line shows the expected correlation coefficients (Spearman’s ρ) between neutral divergence and recombination rate as a function of split time. Dashed line shows the expected Spearman’s ρ between neutral divergence and McVicker’s *B*-values as a function of split time. Red lines denote the proportion of the divergence due to polymorphism that arose in the ancestral population. Error bars denote ± one standard error of the mean. Panels A-D denote different ancestral population sizes (*N*_*a*_). Note that the correlations are greater than 0 for a range of split times and ancestral population sizes, even when the proportion of divergence due to ancestral polymorphism is low.
(PDF)

S5 Fig Variance of the total divergence attributable to the variance in levels of ancestral polymorphism. Black lines show the ratio of the variance of divergence in the ancestral population to the variance of the total divergence as a function of split time. Red lines denote the proportion of the divergence due to polymorphism that arose in the ancestral population. Panels A-D denote different ancestral population sizes (*N*_*a*_).
(PDF)

S6 Fig Correlations between human-primate divergence and genomic features persist when filtering the 25% of the genome with the highest GERP scores. (A) Neutral human-chimp divergence shows a negative correlation with functional content. (B) Neutral human-orang divergence shows a negative correlation with functional content. (C) Neutral human-chimp divergence shows a positive correlation with human recombination rate. (D) Neutral human-orang divergence shows a positive correlation with human recombination rate. Each point represents the mean divergence and functional content (A and B) or recombination rate (C and D) in 1% of the 100kb windows binned by functional content or recombination rate. Red lines indicate the loess curves fit to divergence and functional content (A and B) and divergence and recombination rate (C and D). Note that the last bin containing less than 1% of the windows was omitted from the plot. While the graph presents binned data, the correlations reported in the text are from the unbinned data.
(PDF)

S7 Fig Correlations between human-rodent divergence and genomic features change when filtering the 25% of the genome with the highest GERP scores. (A) Neutral human-mouse divergence no longer correlates with functional content. (B) Neutral human-rat divergence does not correlate with functional content. (C) Neutral human-mouse divergence shows a positive correlation with McVicker’s *B*-values. (D) Neutral human-rat divergence shows a positive correlation with McVicker’s *B*-values. Each point represents the mean divergence and functional content (A and B) or *B*-values (C and D) in 1% of the 100kb windows binned by functional content or *B*-values. Red lines indicate the loess curves fit to divergence and functional content (A and B) and divergence and *B*-values (C and D). Note that the last bin containing less than 1% of the windows was omitted from the plot. While the graph presents binned data, the correlations reported in the text are from the unbinned data.
(PDF)

S8 Fig Observed and modeled genome-wide distributions of human-chimp divergence and human-mouse divergence in 100kb windows when filtering sites whose GERP scores fall into the top 25% of the genome-wide distribution. Gray lines denote 500 simulated genome-wide distributions of divergence. Red line denotes the observed distribution of neutral divergence. Note, the distribution of simulated divergence is comparable to that from empirical data. (A) Simulated human-chimp divergence without the effects of background selection (BGS). (B) Simulated human-chimp divergence with the effects of background selection. (C) Simulated human-mouse divergence without the effects of background selection. (D) Simulated human-mouse divergence with the effects of background selection. We filtered all AT→GC changes between the human and chimp sequences as they could be affected by biased gene conversion. Thus, the distribution of human-chimp divergence shown here is lower than the overall divergence.
(PDF)

S9 Fig Models incorporating background selection can recapitulate the empirical correlations after removing sites with GERP scores falling in the top 25% of the genome-wide distribution. (A) Models of background selection predict a positive correlation between neutral human-chimp divergence and human recombination rate. Because our model does not include biased gene conversion, the empirical correlation was calculated omitting AT to GC sequence differences. (B) Models of background selection predict a positive correlation between neutral human-mouse divergence and McVicker’s *B*-values. The white histogram denotes 500 simulations without including background selection. The gray histogram denotes 500 simulations incorporating background selection. Red lines represent the correlations computed from the empirical data. Thus, plausible levels of background selection can match the observed correlations when using the most stringent filtering criteria while neutral simulations cannot.
(PDF)

S10 Fig Human-mouse mutational parameters used for the simulations. (PDF)

S1 Table Summary statistics of divergence at four-fold sites and at putatively neutral regions. (PDF)

S2 Table Correlation coefficients of human-primate divergence and functional content. (PDF)

S3 Table Correlation coefficients of human-primate divergence and recombination rate. (PDF)

S4 Table Correlation coefficients of human-rodent divergence and functional content. (PDF)

S5 Table Correlation coefficients of human-rodent divergence and McVicker’s *B*-values. (PDF)

S6 Table Summary of parameters used for the coalescent simulations. (PDF)

S7 Table Comparison of the mean and standard deviation of the empirical and simulated divergence. (PDF)

S8 Table GERP RS score cutoff. (PDF)
