# Supplementary figures and images for: Determining the Effect of Natural Selection on Linked Neutral Divergence across Species
Source: PLoS Genet. 2016 Aug 10;12(8):e1006199. doi: 10.1371/journal.pgen.1006199 (PMC4980041; doi:10.1371/journal.pgen.1006199)

**A**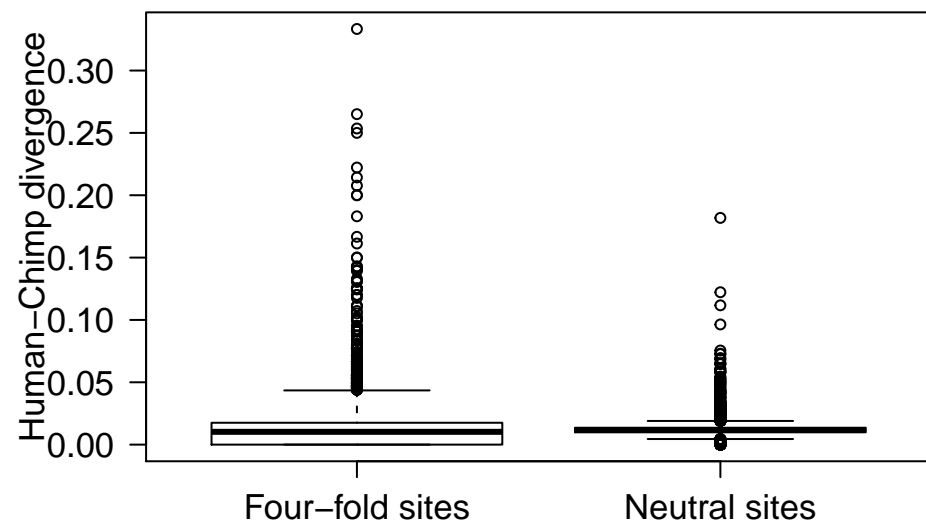**B**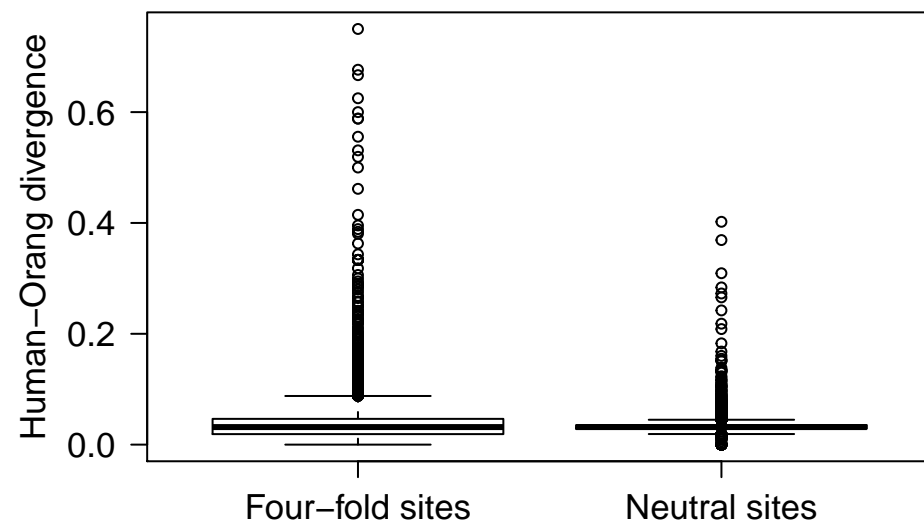**C**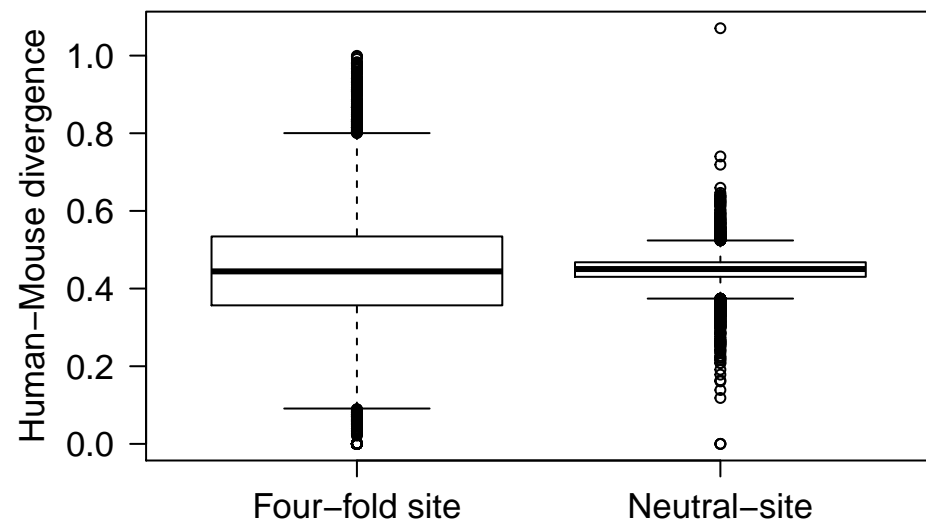**D**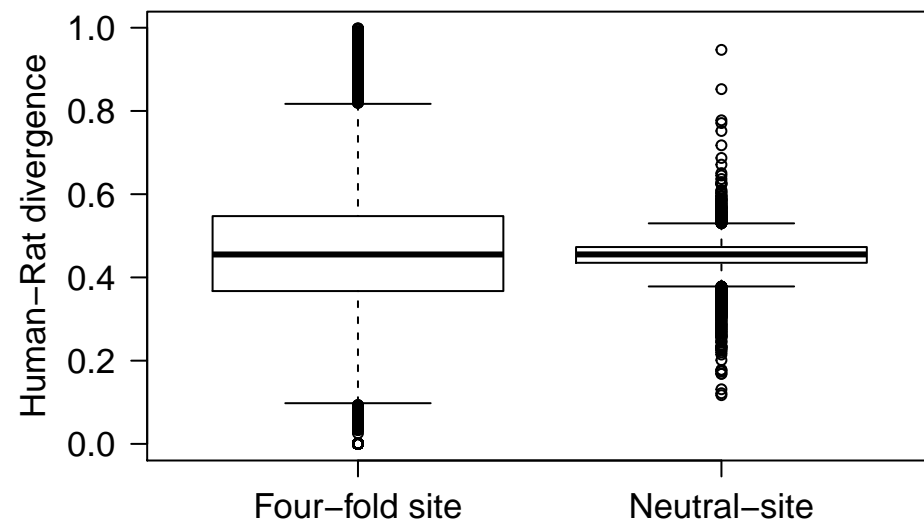

Supplement: S1 Fig — Each point represents the divergence within a 100kb window. (A) Human-chimpanzee, (B) Human-orangutan, (C) Human-mouse, and (D) Human-rat. (PDF) [file pgen.1006199.s001.pdf]

## Divergence & recombination

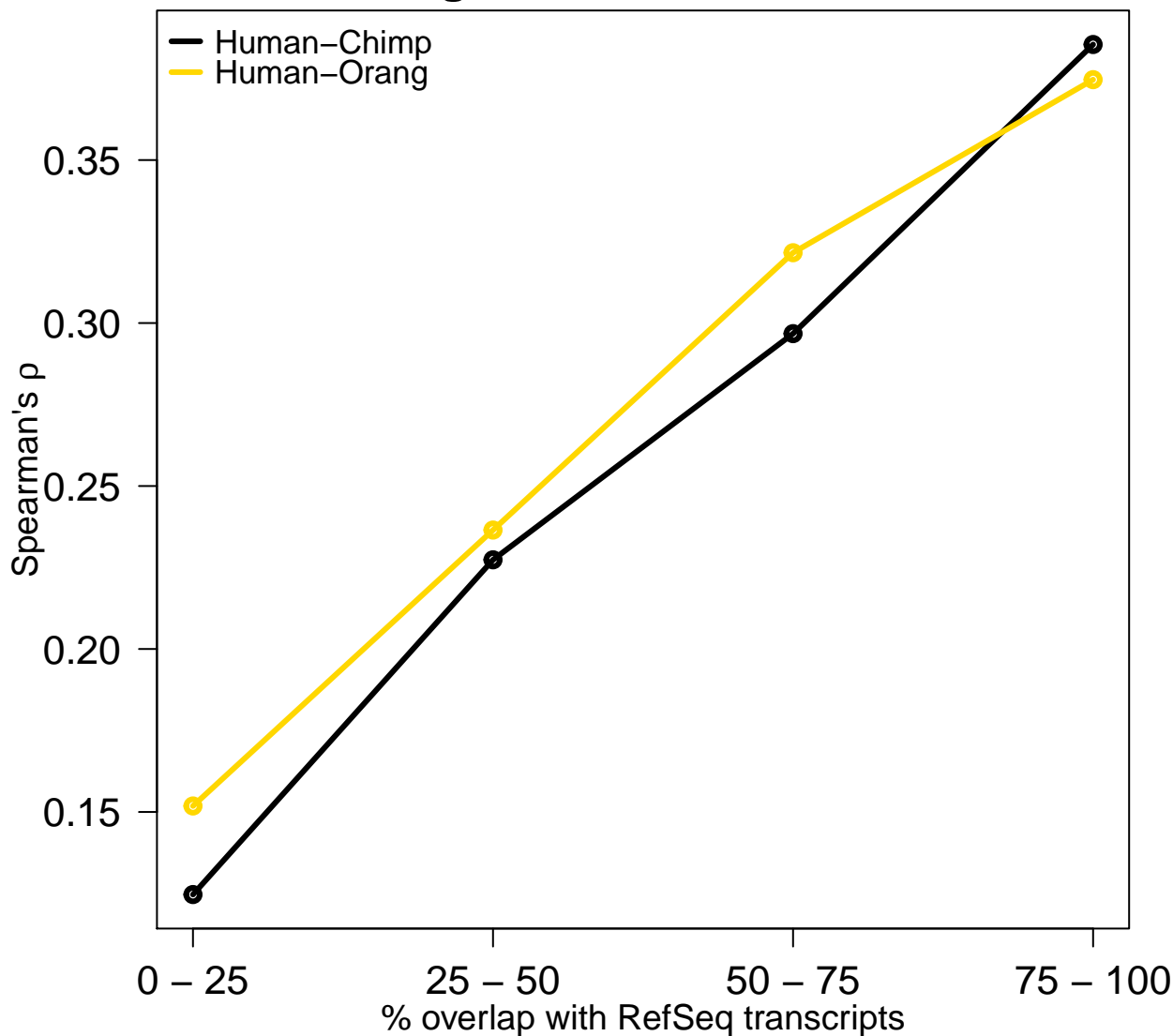

Supplement: S2 Fig — Correlation (Spearman’s ρ) between neutral divergence and human recombination as a function of the amount of overlap with a RefSeq transcript. Black line denotes the correlations between human-chimpanzee neutral divergence and human recombination rate. Yellow line denotes the correlations between human-orangutan neutral divergence and human recombination rate. (PDF) [file pgen.1006199.s002.pdf]

**A. Human–Chimp without BGS**

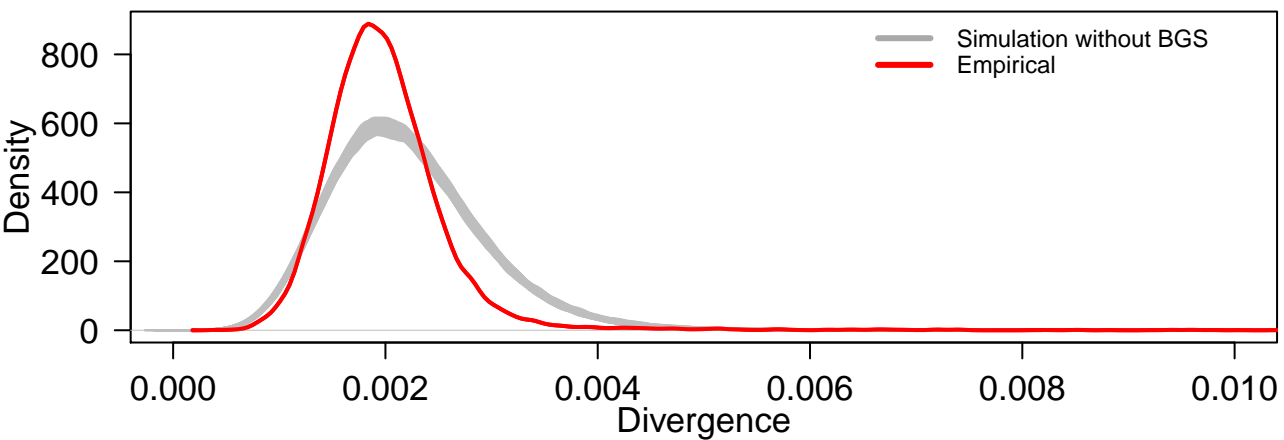

**B. Human–Chimp with BGS**

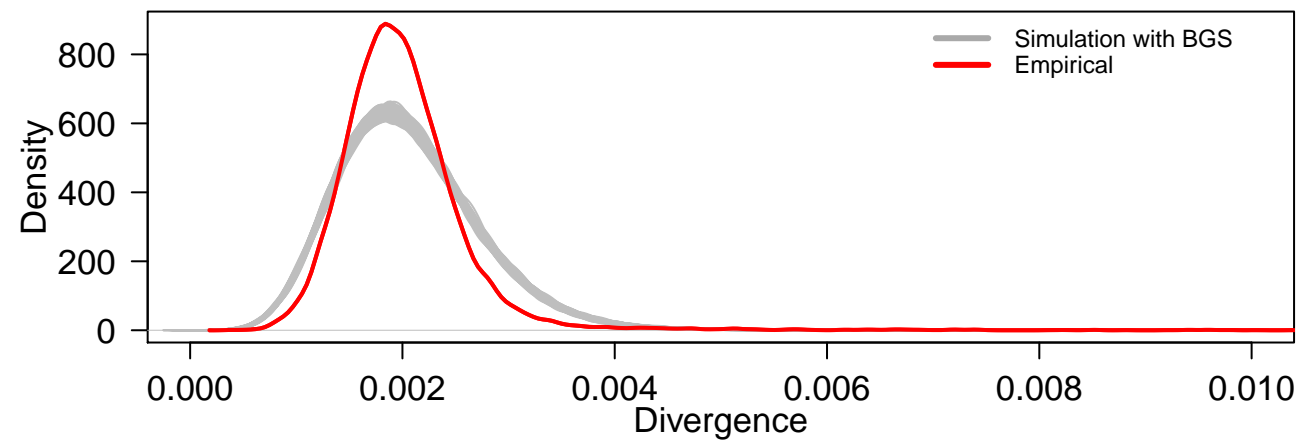

**C. Human–Mouse without BGS**

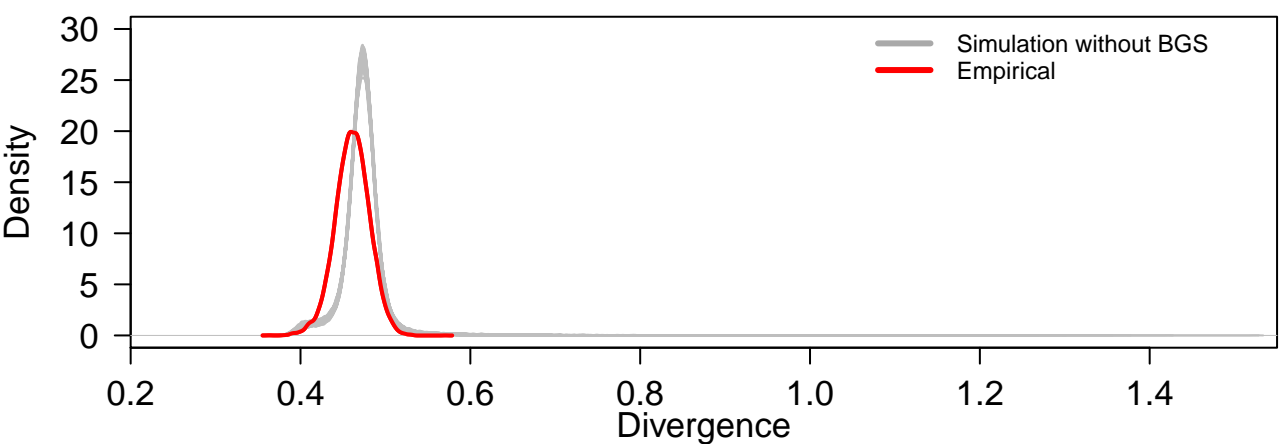

**D. Human–Mouse with BGS**

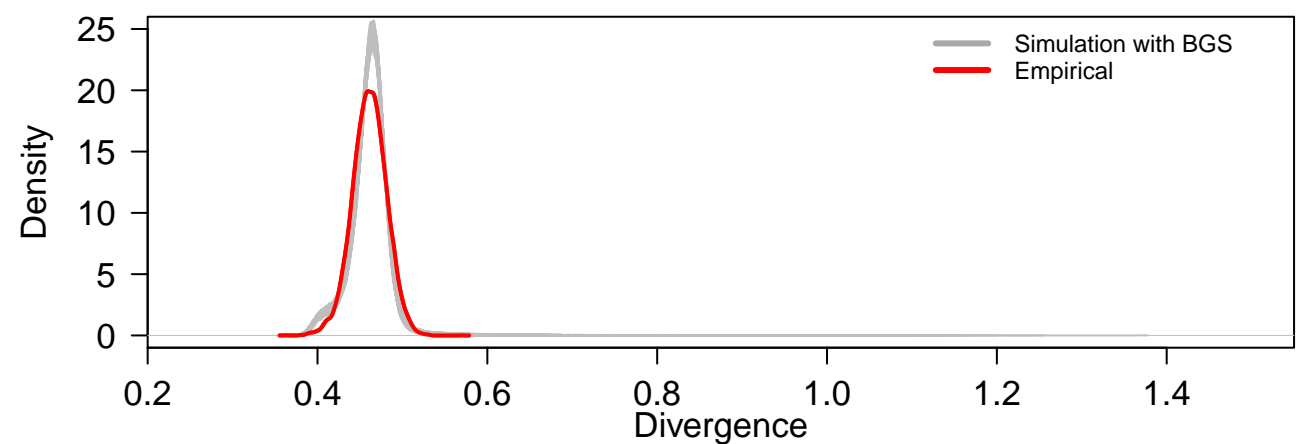

Supplement: S3 Fig — Gray lines denote 500 simulated genome-wide distributions of divergence. Red line denotes the observed distribution of neutral divergence. Note, the distribution of simulated divergence is comparable to that from empirical data. (A) Simulated human-chimp divergence without the effects of background selection (BGS). (B) Simulated human-chimp divergence with the effects of background selection. (C) Simulated human-mouse divergence without the effects of background selection. (D) Simulated human-mouse divergence with the effects of background selection. We filtered all AT→GC changes between the human and chimp sequences as they could be affected by biased gene conversion. Thus, the distribution of human-chimp divergence shown here is lower than the overall divergence. (PDF) [file pgen.1006199.s003.pdf]

**A** $N_a = 25,000$ 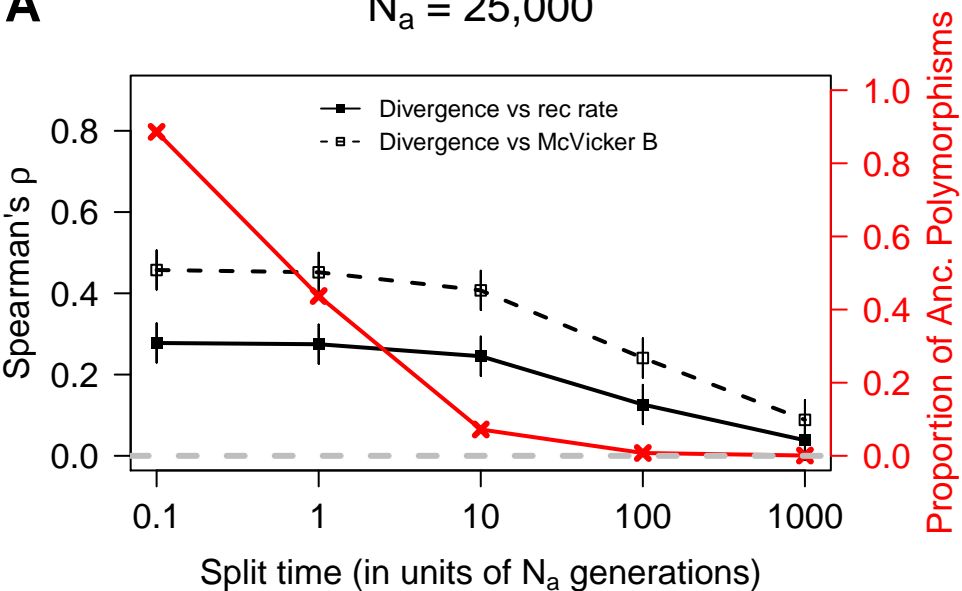**B** $N_a = 50,000$ 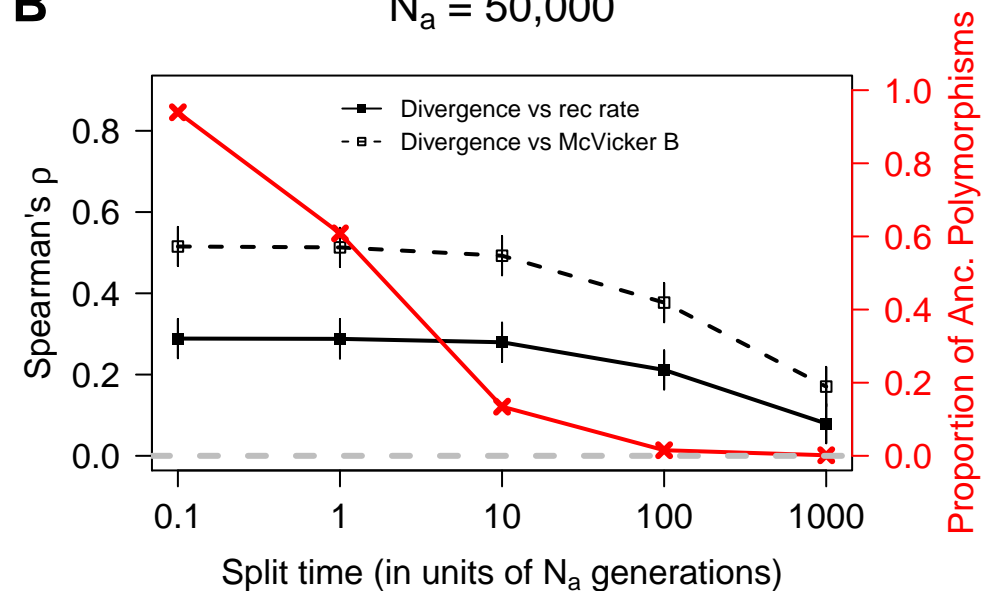**C** $N_a = 100,000$ 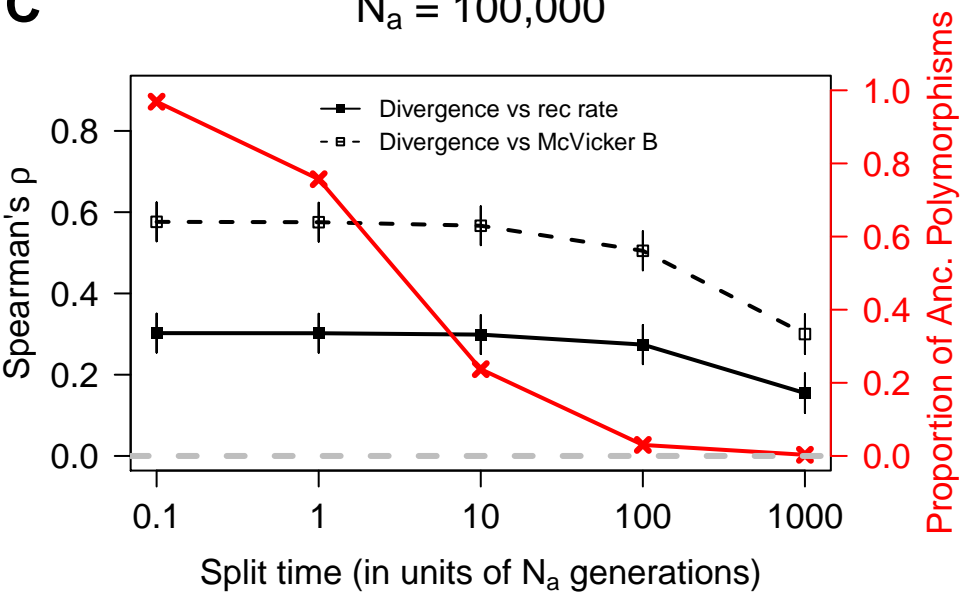**D** $N_a = 200,000$ 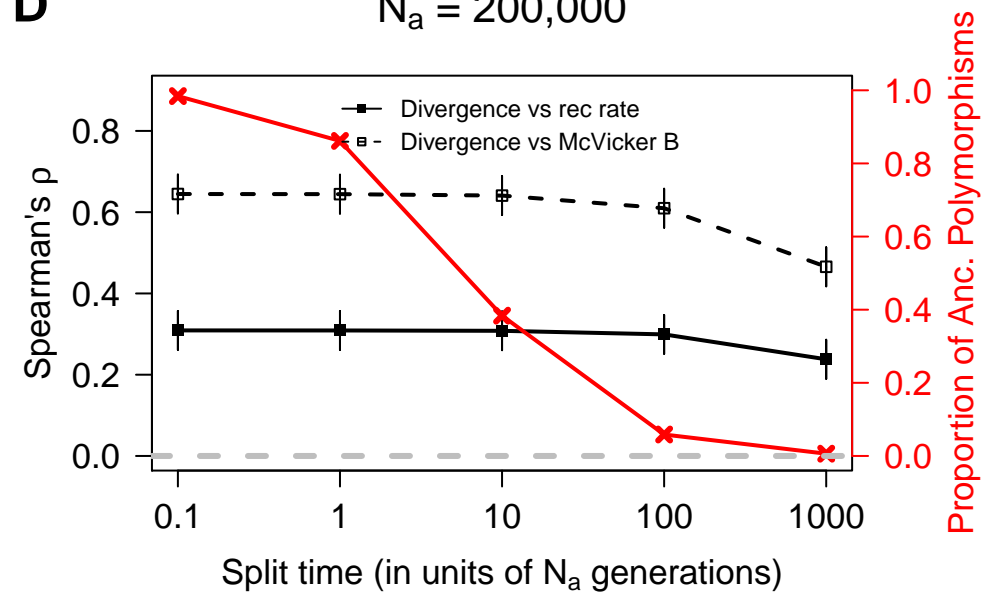

Supplement: S4 Fig — Solid line shows the expected correlation coefficients (Spearman’s ρ) between neutral divergence and recombination rate as a function of split time. Dashed line shows the expected Spearman’s ρ between neutral divergence and McVicker’s B-values as a function of split time. Red lines denote the proportion of the divergence due to polymorphism that arose in the ancestral population. Error bars denote ± one standard error of the mean. Panels A-D denote different ancestral population sizes (Na). Note that the correlations are greater than 0 for a range of split times and ancestral population sizes, even when the proportion of divergence due to ancestral polymorphism is low. (PDF) [file pgen.1006199.s004.pdf]

**A** $N_a = 25,000$ 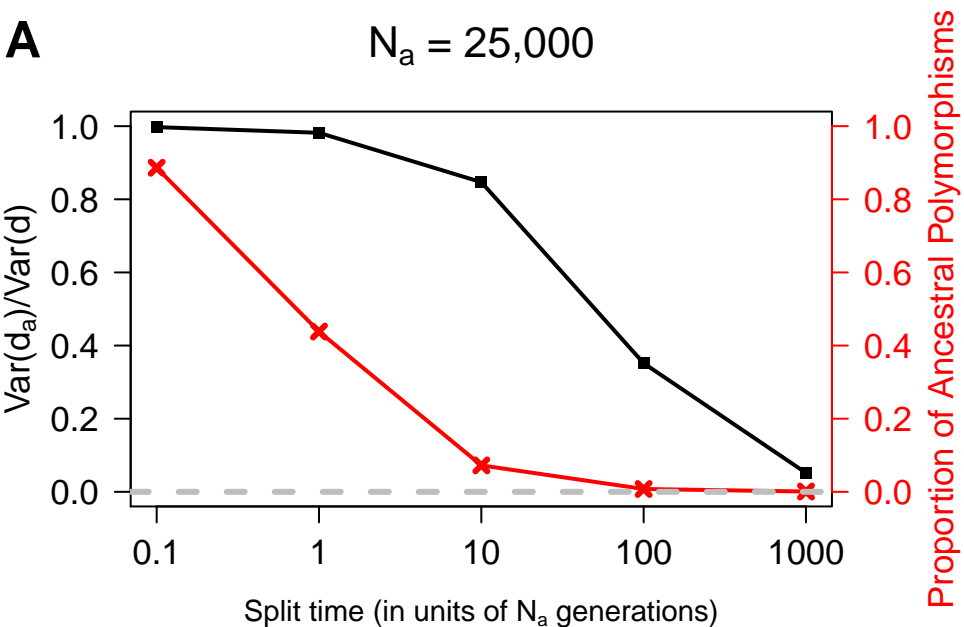**B** $N_a = 50,000$ 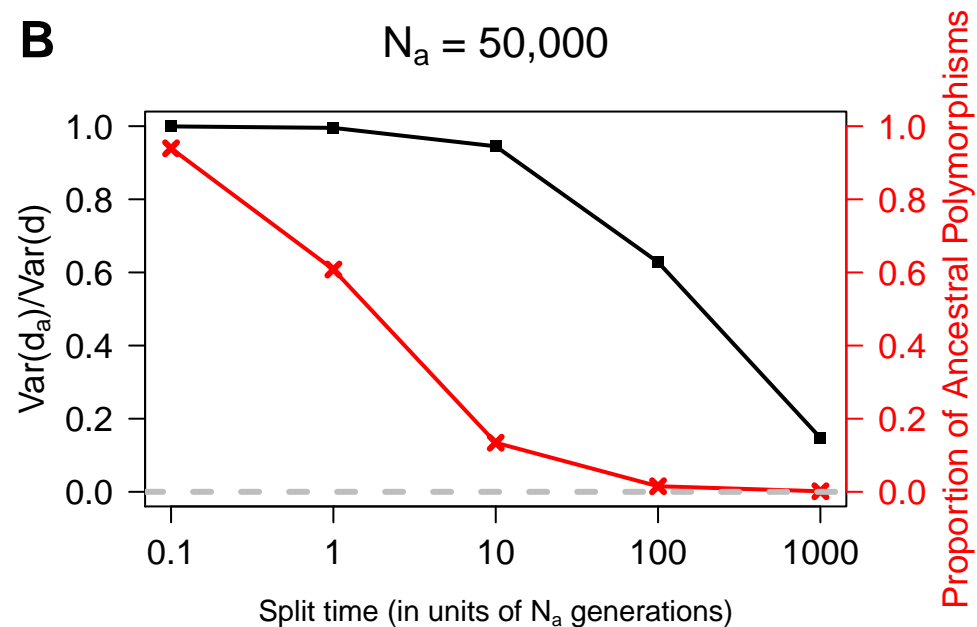**C** $N_a = 100,000$ 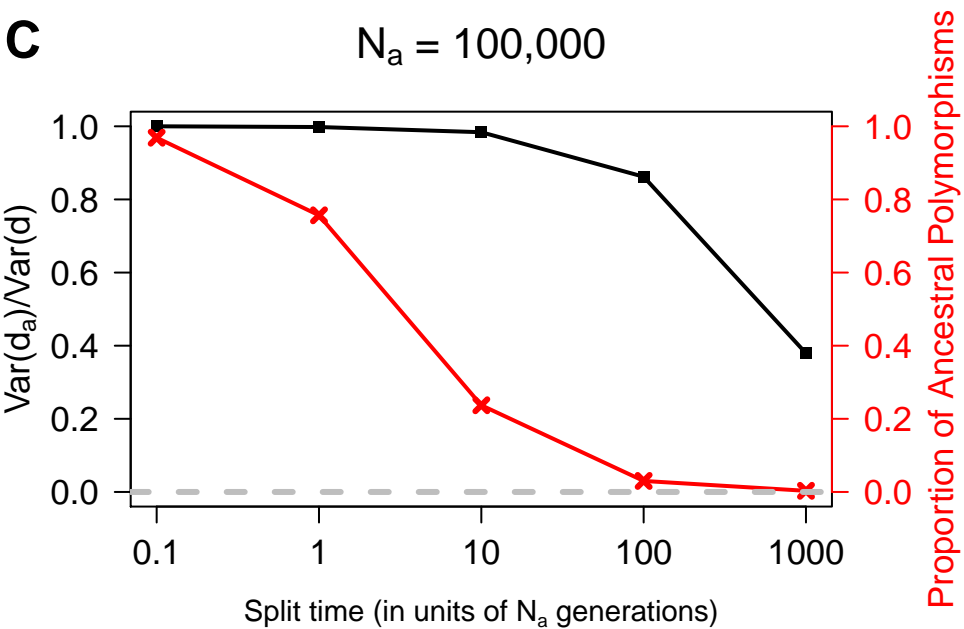**D** $N_a = 200,000$ 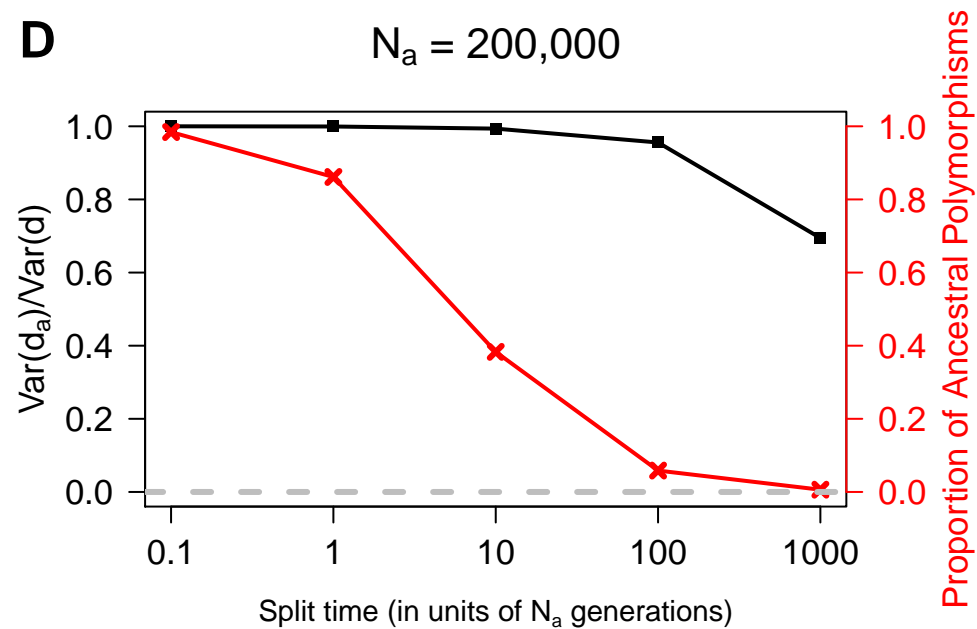

Supplement: S5 Fig — Black lines show the ratio of the variance of divergence in the ancestral population to the variance of the total divergence as a function of split time. Red lines denote the proportion of the divergence due to polymorphism that arose in the ancestral population. Panels A-D denote different ancestral population sizes (Na). (PDF) [file pgen.1006199.s005.pdf]

**A**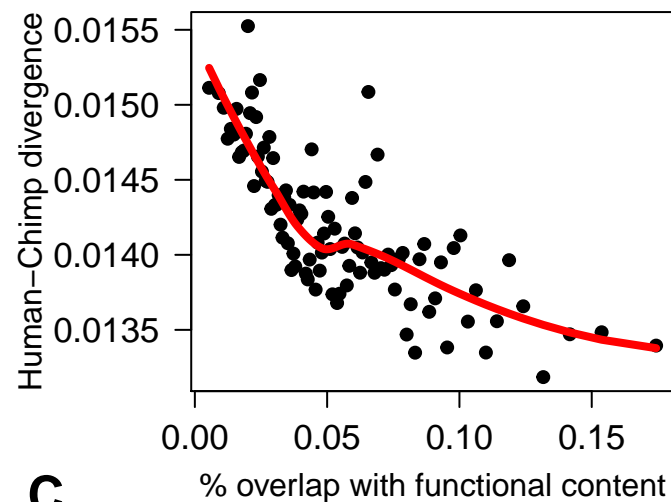**B**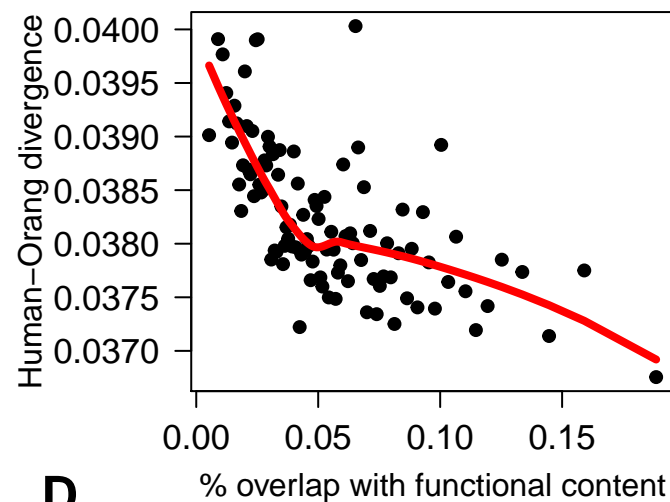**C**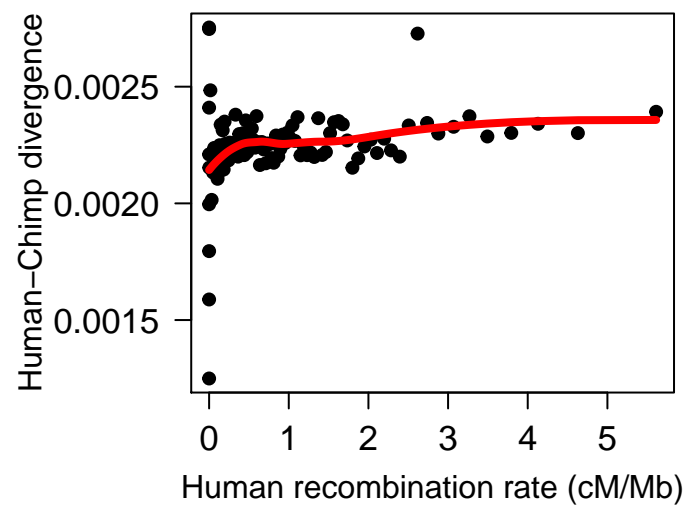**D**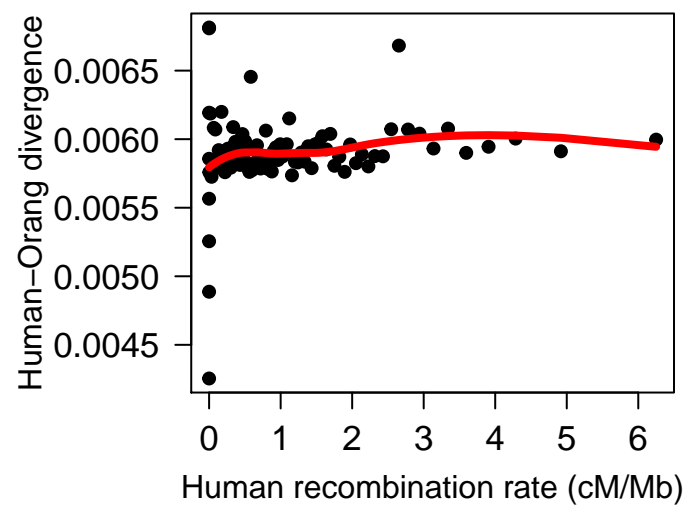

Supplement: S6 Fig — (A) Neutral human-chimp divergence shows a negative correlation with functional content. (B) Neutral human-orang divergence shows a negative correlation with functional content. (C) Neutral human-chimp divergence shows a positive correlation with human recombination rate. (D) Neutral human-orang divergence shows a positive correlation with human recombination rate. Each point represents the mean divergence and functional content (A and B) or recombination rate (C and D) in 1% of the 100kb windows binned by functional content or recombination rate. Red lines indicate the loess curves fit to divergence and functional content (A and B) and divergence and recombination rate (C and D). Note that the last bin containing less than 1% of the windows was omitted from the plot. While the graph presents binned data, the correlations reported in the text are from the unbinned data. (PDF) [file pgen.1006199.s006.pdf]

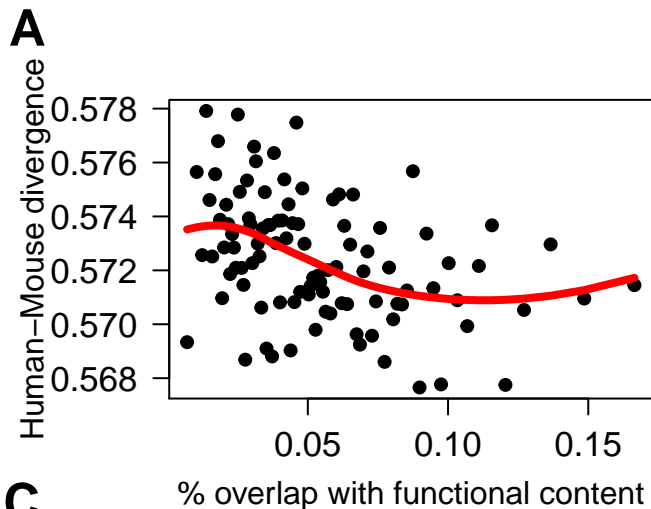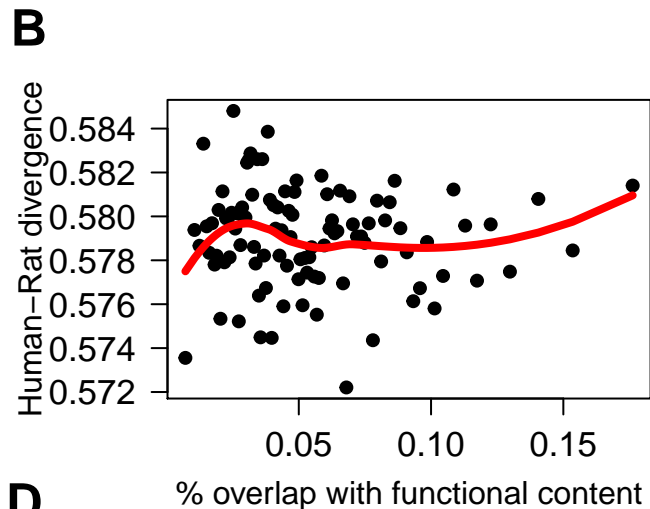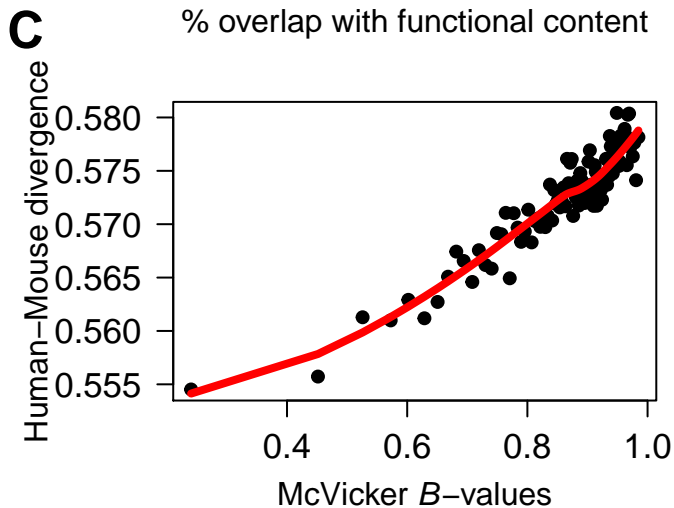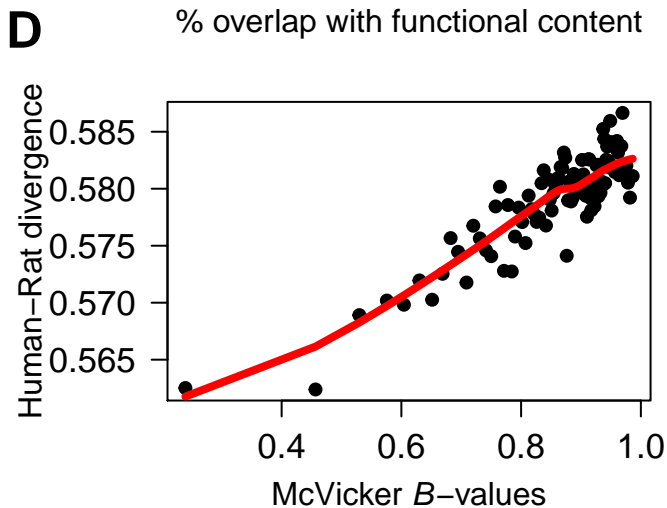

Supplement: S7 Fig — (A) Neutral human-mouse divergence no longer correlates with functional content. (B) Neutral human-rat divergence does not correlate with functional content. (C) Neutral human-mouse divergence shows a positive correlation with McVicker’s B-values. (D) Neutral human-rat divergence shows a positive correlation with McVicker’s B-values. Each point represents the mean divergence and functional content (A and B) or B-values (C and D) in 1% of the 100kb windows binned by functional content or B-values. Red lines indicate the loess curves fit to divergence and functional content (A and B) and divergence and B-values (C and D). Note that the last bin containing less than 1% of the windows was omitted from the plot. While the graph presents binned data, the correlations reported in the text are from the unbinned data. (PDF) [file pgen.1006199.s007.pdf]

**A. Human–Chimp without BGS**

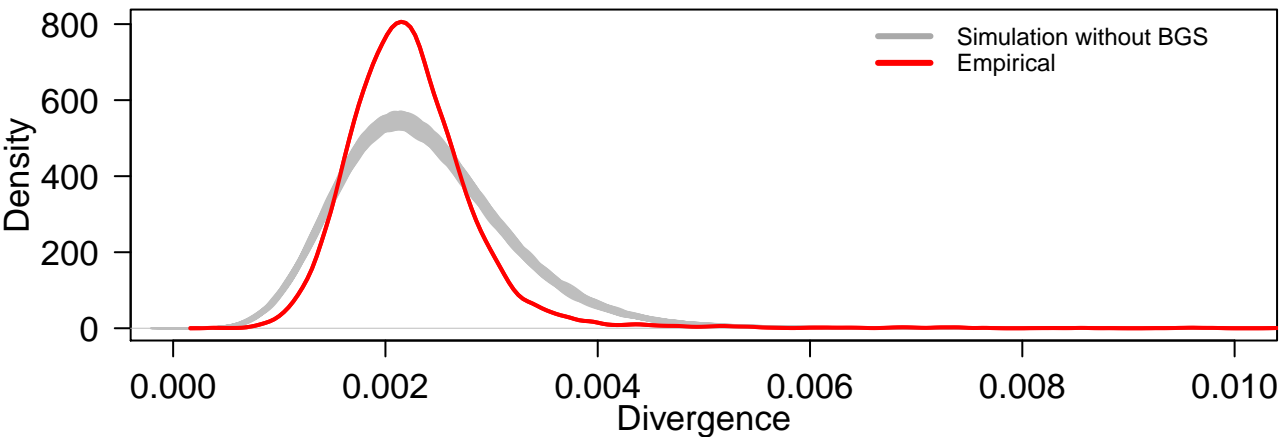

**B. Human–Chimp with BGS**

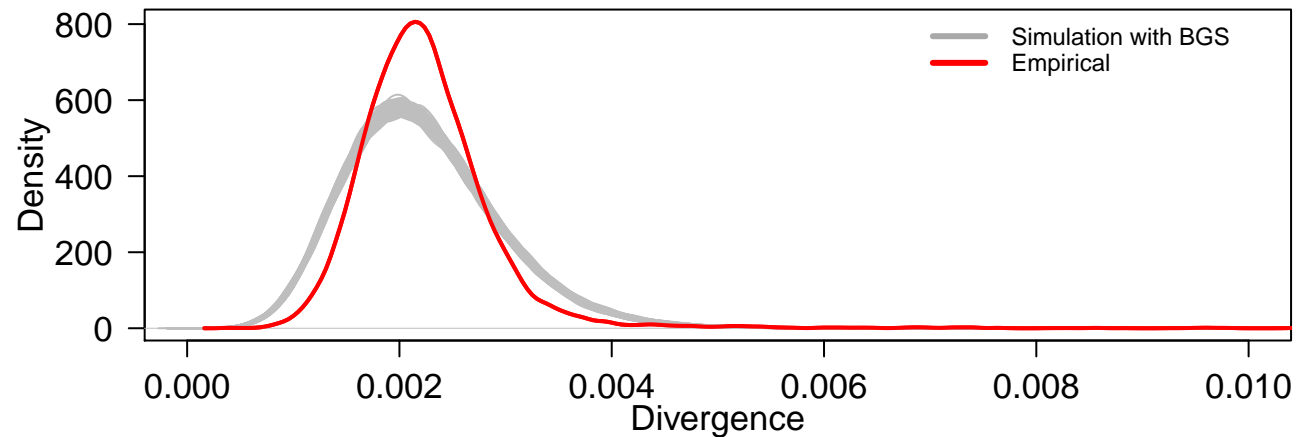

**C. Human–Mouse without BGS**

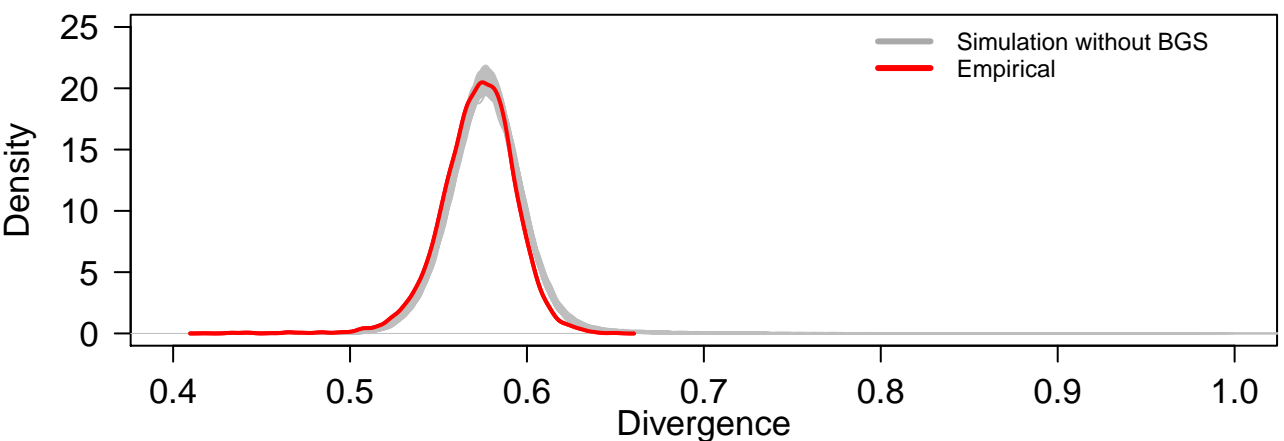

**D. Human–Mouse with BGS**

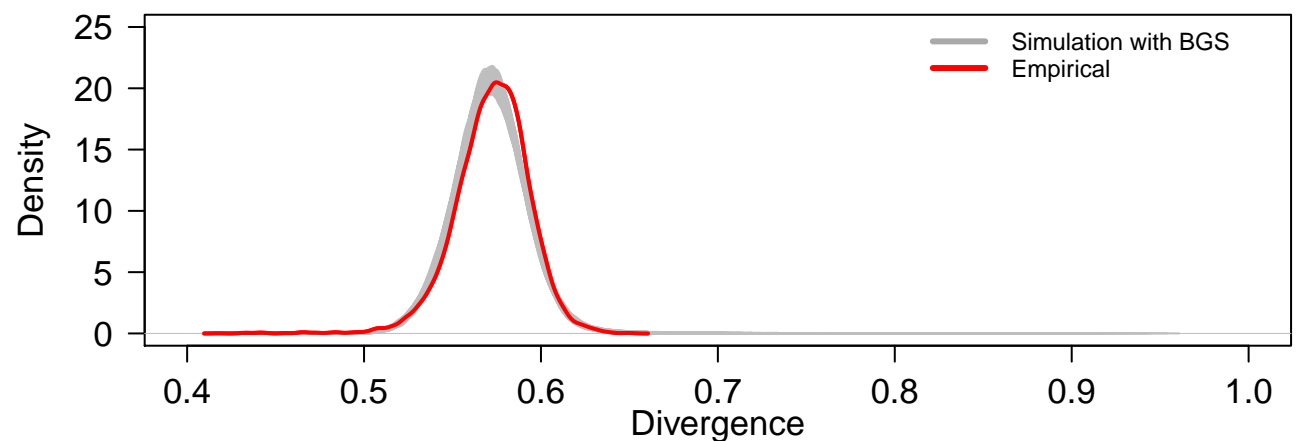

Supplement: S8 Fig — Gray lines denote 500 simulated genome-wide distributions of divergence. Red line denotes the observed distribution of neutral divergence. Note, the distribution of simulated divergence is comparable to that from empirical data. (A) Simulated human-chimp divergence without the effects of background selection (BGS). (B) Simulated human-chimp divergence with the effects of background selection. (C) Simulated human-mouse divergence without the effects of background selection. (D) Simulated human-mouse divergence with the effects of background selection. We filtered all AT→GC changes between the human and chimp sequences as they could be affected by biased gene conversion. Thus, the distribution of human-chimp divergence shown here is lower than the overall divergence. (PDF) [file pgen.1006199.s008.pdf]

**A. Human–Chimp GERP 25%**

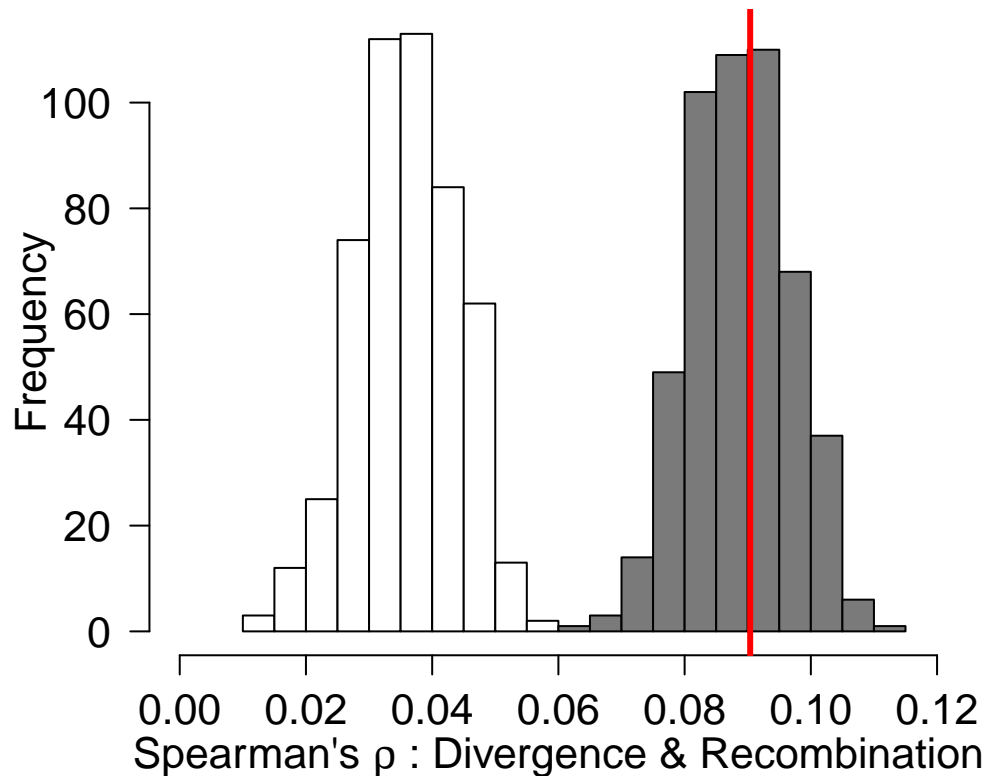

**B. Human–Mouse GERP 25%**

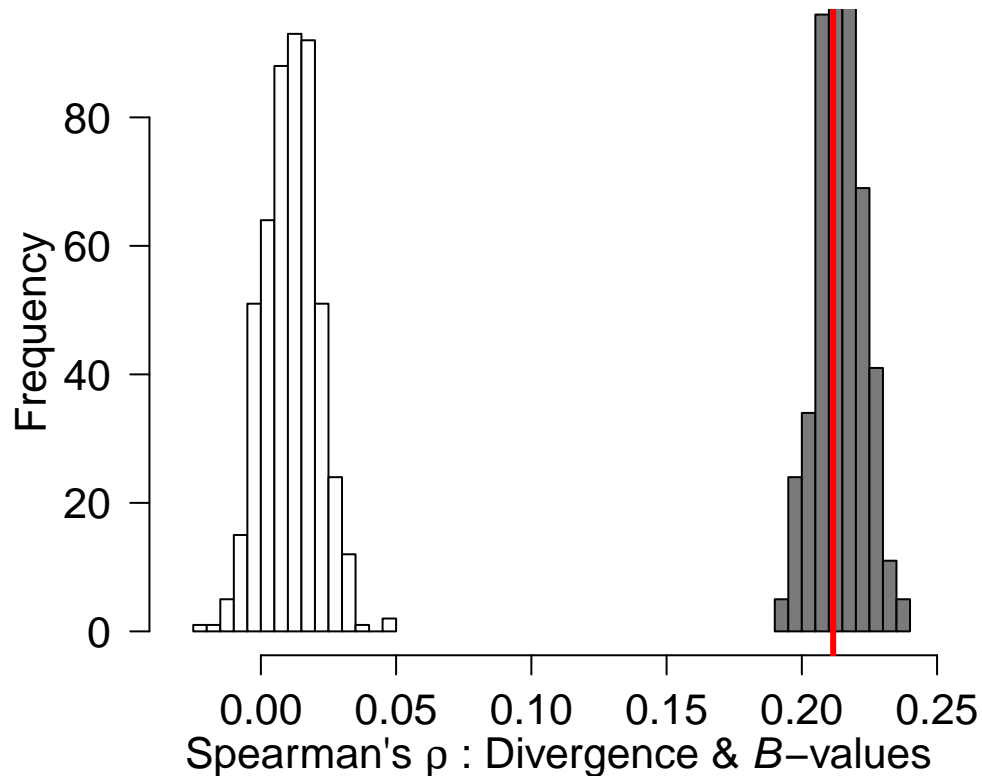

Supplement: S9 Fig — (A) Models of background selection predict a positive correlation between neutral human-chimp divergence and human recombination rate. Because our model does not include biased gene conversion, the empirical correlation was calculated omitting AT to GC sequence differences. (B) Models of background selection predict a positive correlation between neutral human-mouse divergence and McVicker’s B-values. The white histogram denotes 500 simulations without including background selection. The gray histogram denotes 500 simulations incorporating background selection. Red lines represent the correlations computed from the empirical data. Thus, plausible levels of background selection can match the observed correlations when using the most stringent filtering criteria while neutral simulations cannot. (PDF) [file pgen.1006199.s009.pdf]
