# Supplementary material for: Determining the Effect of Natural Selection on Linked Neutral Divergence across Species
Source: PLoS Genet. 2016 Aug 10;12(8):e1006199. doi: 10.1371/journal.pgen.1006199 (PMC4980041; doi:10.1371/journal.pgen.1006199)
Supplement: S10 Fig — (PDF) [file pgen.1006199.s010.pdf]

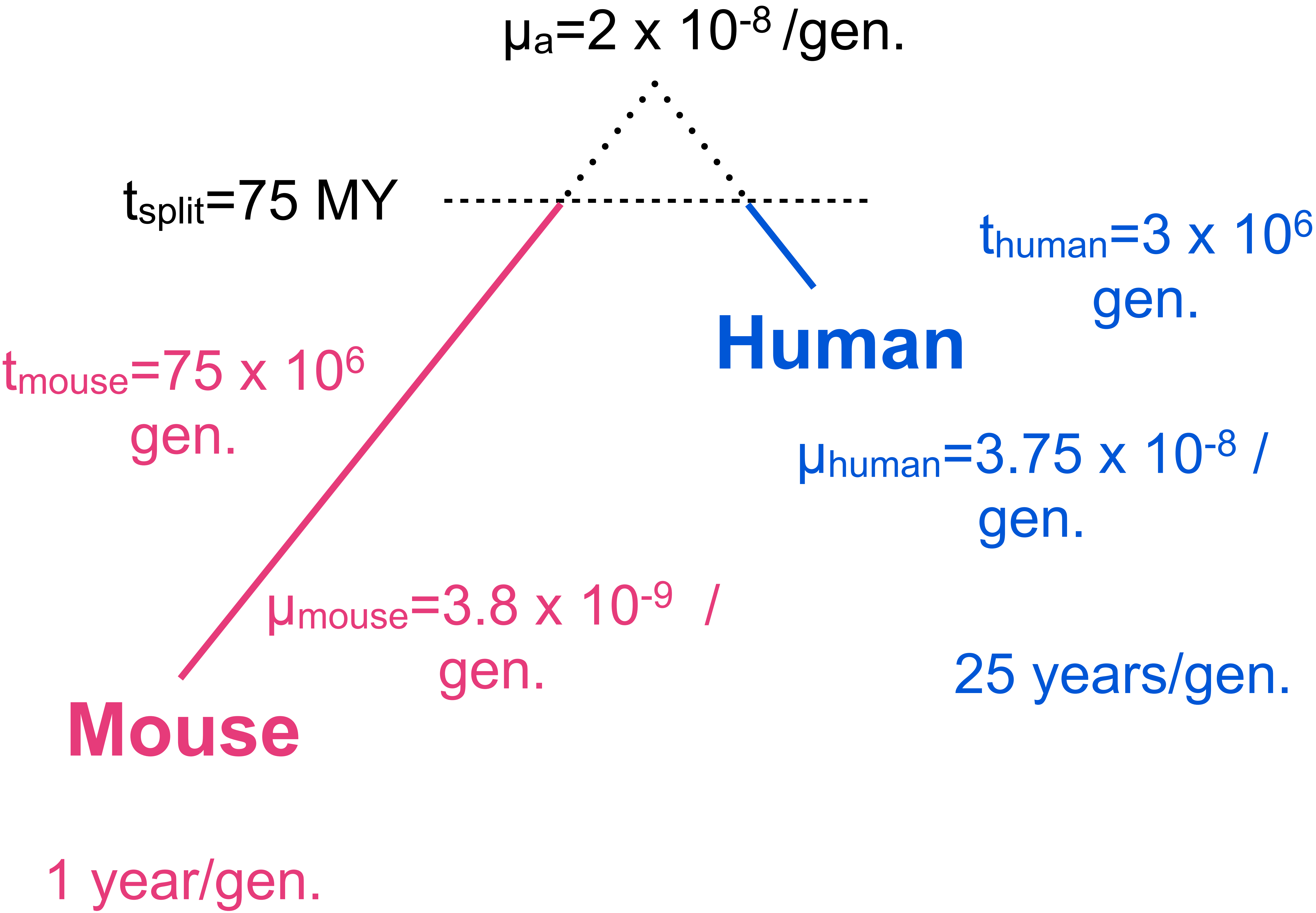
$\mu_a = 2 \times 10^{-8}$ /gen.
$t_{split} = 75$ MY
$t_{human} = 3 \times 10^6$ gen.
Human
$t_{mouse} = 75 \times 10^6$ gen.
$\mu_{human} = 3.75 \times 10^{-8}$ / gen.
$\mu_{mouse} = 3.8 \times 10^{-9}$ / gen.
25 years/gen.
Mouse
1 year/gen.
