# Supplementary material for: Determining the Effect of Natural Selection on Linked Neutral Divergence across Species
Source: PLoS Genet. 2016 Aug 10;12(8):e1006199. doi: 10.1371/journal.pgen.1006199 (PMC4980041; doi:10.1371/journal.pgen.1006199)
Supplement: S1 Table — (PDF) [file pgen.1006199.s011.pdf]

S1 Table: Summary statistics of divergence at four-fold sites and at putatively neutral regions

Species pair		Mean of divergence	Standard deviation of divergence
Human-chimp	Four-fold sites	0.013	0.016
	Putatively neutral sites	0.012	0.005
Human-orang	Four-fold sites	0.037	0.037
	Putatively neutral sites	0.033	0.012
Human-mouse	Four-fold sites	0.444	0.152
	Putatively neutral sites	0.447	0.038
Human-rat	Four-fold sites	0.456	0.156
	Putatively neutral sites	0.452	0.039

The mean and standard deviation of divergence were computed over 100kb windows at four-fold degenerate sites and at putatively neutral sites. Windows where the total number of eligible sites was equal to 0 were excluded from both sets. Further, for human-mouse and human-rat, we corrected for multiple mutations with Kimura 2-parameter models. Note that for human-mouse and human-rat, we also excluded windows with unrealistically high divergence after the Kimura 2-parameter correction (i.e. windows where the divergence was greater than 1 were removed).
