# Supplementary material for: Determining the Effect of Natural Selection on Linked Neutral Divergence across Species
Source: PLoS Genet. 2016 Aug 10;12(8):e1006199. doi: 10.1371/journal.pgen.1006199 (PMC4980041; doi:10.1371/journal.pgen.1006199)
Supplement: S2 Table — (PDF) [file pgen.1006199.s012.pdf]

S2 Table: Correlation coefficients of human-primate divergence and functional content

Species pair	Spearman's ρ overall	Spearman's ρ post CpG filtering	Partial correlation controlling for GC content	Spearman's ρ post gBGC filtering[a]	Partial correlation controlling for recombination	Spearman's ρ when using 50kb-windows
Human-chimp	-0.235**	-0.252**	-0.291**	-0.243**	-0.269**	-0.186**
Human-orang	-0.204**	-0.232**	-0.289**	-0.255**	-0.241**	-0.184**

**p-value < 2.2e-16

[a]Spearman's ρ after filtering sites possibly affected by GC-biased gene conversion (see text).
