# Supplementary material for: Determining the Effect of Natural Selection on Linked Neutral Divergence across Species
Source: PLoS Genet. 2016 Aug 10;12(8):e1006199. doi: 10.1371/journal.pgen.1006199 (PMC4980041; doi:10.1371/journal.pgen.1006199)
Supplement: S3 Table — (PDF) [file pgen.1006199.s013.pdf]

S3 Table: Correlation coefficients of human-primate divergence and recombination rate.

Species pair	Spearman's ρ overall	Spearman's ρ post CpG filtering	Partial correlation controlling for GC content	Spearman's ρ post gBGC filtering[a]	Spearman's ρ when using 50 kb windows
Human-chimp	0.234**	0.207**	0.242**	0.108**	0.182**
Human-orang	0.249**	0.221**	0.240**	0.088**	0.209**

**p-value < 2.2e-16

[a]Spearman's ρ after filtering sites possibly affected by GC-biased gene conversion (see text).
