# Supplementary material for: Determining the Effect of Natural Selection on Linked Neutral Divergence across Species
Source: PLoS Genet. 2016 Aug 10;12(8):e1006199. doi: 10.1371/journal.pgen.1006199 (PMC4980041; doi:10.1371/journal.pgen.1006199)
Supplement: S4 Table — (PDF) [file pgen.1006199.s014.pdf]

S4 Table: Correlation coefficients of human-rodent divergence and functional content.

Species pair	Spearman's ρ overall	Spearman's ρ post CpG filtering	Partial correlation controlling for GC content	Partial correlation controlling for recombination	Spearman's ρ post gBGC filtering[a]	Spearman's ρ when using 50 kb windows
Human-mouse	-0.184**	-0.202**	-0.243**	-0.194**	-0.339**	-0.135**
Human-rat	-0.149**	-0.169**	-0.206**	-0.156**	-0.337**	-0.103**

**p-value < 2.2e-16

[a]Spearman's ρ after filtering sites possibly affected by GC-biased gene conversion (see text).
