# Supplementary material for: Determining the Effect of Natural Selection on Linked Neutral Divergence across Species
Source: PLoS Genet. 2016 Aug 10;12(8):e1006199. doi: 10.1371/journal.pgen.1006199 (PMC4980041; doi:10.1371/journal.pgen.1006199)
Supplement: S5 Table — (PDF) [file pgen.1006199.s015.pdf]

S5 Table: Correlation coefficients of human-rodent divergence and McVicker's *B*-values

Species pair	Spearman's ρ overall	Spearman's ρ post CpG filtering	Partial correlation controlling for GC content	Spearman's ρ post gBGC filtering[a]	Spearman's ρ when using 50 kb windows
Human-mouse	0.445**	0.450**	0.456**	0.419**	0.403**
Human-rat	0.402**	0.405**	0.413**	0.404**	0.366**

**p-value < 2.2e-16

[a]Spearman's ρ after filtering sites possibly affected by GC-biased gene conversion (see text).
