# Supplementary material for: Determining the Effect of Natural Selection on Linked Neutral Divergence across Species
Source: PLoS Genet. 2016 Aug 10;12(8):e1006199. doi: 10.1371/journal.pgen.1006199 (PMC4980041; doi:10.1371/journal.pgen.1006199)
Supplement: S6 Table — (PDF) [file pgen.1006199.s016.pdf]

**S6 Table:** Summary of parameters used for the coalescent simulations.

| Species pair | Shape | Scale | μ (average)[a] | $N_a$ | t-split (generations) |
|---|---|---|---|---|---|
| Human-chimp GERP 10 | 16.82 | $1.7X10^{-10}$ | $2.9X10^{-9}$ | 70000 | 240000 |
| Human-chimp GERP 25 | 15.68 | $1.8X10^{-10}$ | $2.8X10^{-9}$ | 71000 | 280000 |
| Human-mouse GERP 10 | $4.0X10^{3}$ | $5.0X10^{-12}$ | $2.0X10^{-8}$ | 940000 | N/A |
| Human-mouse GERP 25 | $1.6X10^{3}$ | $1.7X10^{-11}$ | $2.7X10^{-8}$ | 375000 | N/A |

Note that shape and scale refer to the shape and scale parameters used for the gamma distribution of mutation rates.

[a]We filtered all AT→GC changes between the human and chimp sequences as they could be affected by biased gene conversion. Thus, the mutation rates used here are substantially lower than commonly used values.
