# Supplementary material for: Determining the Effect of Natural Selection on Linked Neutral Divergence across Species
Source: PLoS Genet. 2016 Aug 10;12(8):e1006199. doi: 10.1371/journal.pgen.1006199 (PMC4980041; doi:10.1371/journal.pgen.1006199)
Supplement: S7 Table — (PDF) [file pgen.1006199.s017.pdf]

S7 Table: Comparison of the mean and standard deviation of the empirical and simulated divergence

Species pair	Empirical mean	Average of the mean of simulated divergence from 500 simulations		Empirical standard deviation	Average of the standard deviation of simulated divergence from 500 simulations	
		Without BGS	With BGS		Without BGS	With BGS
Human-chimp GERP 10	0.002	0.002[a]	0.002[a]	$6.8X10^{-4}$	$7.4X10^{-4}$	$6.7X10^{-4}$
Human-chimp GERP 25	0.002	0.002[a]	0.002[a]	$7.2X10^{-4}$	$7.9X10^{-4}$	$7.3X10^{-4}$
Human-mouse GERP 10	0.461	0.473	0.461	0.020	0.027	0.024
Human-mouse GERP 25	0.572	0.577	0.571	0.021	0.022	0.021

Note that empirical mean and empirical standard deviation refer to the average neutral divergence calculated over 100kb windows.

[a]We filtered all AT→GC changes between the human and chimp sequences as they could be affected by biased gene conversion. Thus, the mean human-chimp divergence reported here is lower than the overall divergence.
