# Supplementary material for: Determining the Effect of Natural Selection on Linked Neutral Divergence across Species
Source: PLoS Genet. 2016 Aug 10;12(8):e1006199. doi: 10.1371/journal.pgen.1006199 (PMC4980041; doi:10.1371/journal.pgen.1006199)
Supplement: S8 Table — (PDF) [file pgen.1006199.s018.pdf]

S8 Table: GERP RS score cutoff

Proportion of the genome to be removed	Remove sites whose RS score is
5%	> 4.949
10%	> 4.078
15%	> 3.207
20%	> 2.336
25%	> 1.465

Note that we also removed sites whose RS score is equal to 0.
